# Construction of a high-density genetic map based on large-scale marker development in *Coix lacryma-jobi* L. using specific-locus amplified fragment sequencing (slaf-seq)

**DOI:** 10.1038/s41598-024-58167-8

**Published:** 2024-04-26

**Authors:** Chenglong Yang, Xiuwen Ban, Mingqiang Zhou, Yu Zhou, Kai Luo, Xiaoyu Yang, Zhifang Li, Fanzhi Liu, Qing Li, Yahong Luo, Xiang Zhou, Jing Lei, Peilin Long, Jian Wang, Jianchun Guo

**Affiliations:** 1 Guizhou Institute of Subtropical Crops, Guizhou Academy of Agricultural Sciences, Xingyi, 542600 Guizhou People’s Republic of China; 2 Key Laboratory of Crop Gene Resources and Germplasm Innovation in Karst Plateau Mountains, Guiyang, 550025 Guizhou People’s Republic of China; 3 The Key Laboratory of Agricultural Bioengineering, Guizhou University, Guiyang, 550025 Guizhou People’s Republic of China; 4 Hainan Institute for Tropical Agricultural Resources & Institute of Tropical Bioscience and Biotechnology, CATAS, Haikou, 571101 People’s Republic of China

**Keywords:** *Coix lacryma-jobi* L., SLAF-seq, High-density genetic map, SNPs, Linkage group, Genetics, Molecular biology, Plant sciences

## Abstract

*Coix lacryma-jobi* L. is one of the most economically and medicinally important corns. This study constructed a high-density genetic linkage map of *C. lacryma-jobi* based on a cross between the parents 'Qianyi No. 2' × 'Wenyi No. 2' and their F_2_ progeny through high-throughput sequencing and the construction of a specific-locus amplified fragment (SLAF) library. After pre-processing, 325.49 GB of raw data containing 1628 M reads were obtained. A total of 22,944 high-quality SLAFs were identified, among which 3952 SLAFs and 3646 polymorphic markers met the requirements for the construction of a genetic linkage map. The integrated map contained 3605 high-quality SLAFs, which were grouped into ten genetic linkage groups. The total length of the map was 1620.39 cM, with an average distance of 0.45 cM and an average of 360.5 markers per linkage group. This report presents the first high-density genetic map of *C. lacryma-jobi*. This map was constructed using an F_2_ population and SLAF-seq approach, which allows the development of a large number of polymorphic markers in a short period. These results provide a platform for precise gene/quantitative trait locus (QTL) mapping, map-based gene separation, and molecular breeding in *C. lacryma-jobi*. They also help identify a target gene for tracking, splitting quantitative traits, and estimating the phenotypic effects of each QTL for QTL mapping. They are of great significance for improving the efficiency of discovering and utilizing excellent gene resources of *C. lacryma-jobi*.

## Introduction

*Coix lacryma-jobi* L. (Job’s tears, medicine corn, myotonin, and six grains) is an annual or perennial C_4_ herb belonging to Maydeae, Gramineae. It is widely grown in East and Southeast Asia^1^. Therefore, Southwestern China is one of the centers for the origin, evolution, and migration^2,3^. It is a traditional crop with high nutritional value and one of the most crucial components of traditional Chinese herbal medicine^4–6^. An application of its seed oil, Kanglaite injection, has been widely used for cancer therapy^7,8^. With the widespread functional recognition of its nutritional value and bioactivities such as anti-tumor, immunomodulation, and blood calcium lowering effects, the demand for Job’s tears has enhanced rapidly as it is widely used as a medicinal and health product in almost all tropical and subtropical countries around the world^2^.

To date, traditional breeding employing domesticated wild resources, hybrids, and mutants has been mainly utilized to develop new varieties of Job’s tears. However, these efforts were limited due to specific inherent characteristics and limitations in breeding techniques. Several economically important traits of Job’s tears are controlled by multiple gene loci. To improve these traits and the breeding efficiency, marker-assisted selection (MAS) and quantitative trait loci (QTLs) can be used to locate and clone the related genes. Genetic maps, especially high-density ones, are essential tools for QTL- and MAS-based research. RAPD was used for the genetic evaluation of the seed resources of Job’s tears^9^. An analysis of the genetic relationships of 79 varieties indicated that the genetic diversities of the varieties in Guangxi, China, were higher than those in South Korea^10^. The genetic relationship of 139 varieties was evaluated using AFLP, which demonstrated that southwestern China was its secondary center of origin^3^. A genetic map of 131 individuals of the F_2_ community derived from a cross between parents from Beijing and Wuhan was built, which included ten genetic clusters, 80 AFLP markers, and ten RFLP markers, totaling 1339.5 cM in length, with an average intergene space of 14.88 cM^11^. The complete genome of the variety ‘Daheishan’ totaling 1.6 Gb was mapped; the genetic linkages of 551 F_2_ individuals from ‘Daheishan’ (male parent) × ‘Xiaobaike’ (female parent) and BC4 backcrossed with ‘Xiaobaike’ was constructed. This map contained 230 InDel markers, with a total length of 1570.12 cM and an average gene spacing of 6.83 cM. This study also accurately identified the genes *Ccph1* and *Ccph2*, which control the thickness and color of the seed shell^12^.

Some genetic maps were constructed by employing molecular marking techniques such as AFLP, RFLP, RAPD, ISSR, SRAP, and SSR. Due to the limited number of individuals used for building these maps, a low number of molecular markers were identified, and the marking densities were not saturated enough, which implied difficulty in carrying out follow-up studies such as using QTLs. Specific-locus amplified fragment sequencing (SLAF-seq) technology is an efficient method of de novo single nucleotide polymorphism (SNP) discovery and large-scale genotyping, which is based on reduced-representation library (RRL) and high-throughput sequencing^13^. SLAF-seq is a powerful tool for genetic research, which was subsequently used to develop SLAF markers in Lophopyrum elongatum^14^ and corn^15^ and for high-density genetic and QTL mapping in sesame seeds^16^, soybeans^17^, and mango^18^. SLAF-seq has therefore become a preferred method for developing highdensity genetic maps for species in the absence of a reference to genome sequences.

Hybrid breeding of *C. lacryma-jobi* was conducted 10 years ago, which established many isolated populations. After years of field investigation, a group of hybrid combinations with significant differences in traits was selected to construct their genetic linkage map. The recently developed SLAF-seq method was used to rapidly identify extensive SNP markers in *C. lacryma-jobi*, thus generating its high-density genetic map and studying its genomic characteristics. The marker development methods utilized in this study and the future applications of this genetic map are discussed.

## Results

### Analysis of SLAF-seq data and SLAF markers

Using two-terminal sequencing of the SLAF library constructed based on SLAF-Seq, a total of 1,628,398,591 reads with ~ 325.49 GB of raw data was obtained (Table S1). The effective length of each read was ~ 384–464 bp post-removal of the label sequences at the ends of the DNA fragments. The average Q30 of the reads obtained by high-throughput sequencing of the 200 F_2_ individuals was ~ 93.92%, and the GC content was 44.43–49.75%, with an average of 46.96%. To improve the efficiency of the molecular markers, the length for SLAF-seq used was much higher for the parents than for the F_2_. Therefore, of the total reads, 66,927,932 originated from the male parent, and 52,144,104 originated from the female parent. In the 200 F_2_ individuals, the sample sequences ranged from 2,878,250 to 14,176,926, with an average value of 7,546,632 (Table S1).

### SLAF polymorphism analysis and genotyping

**Figure 1:**
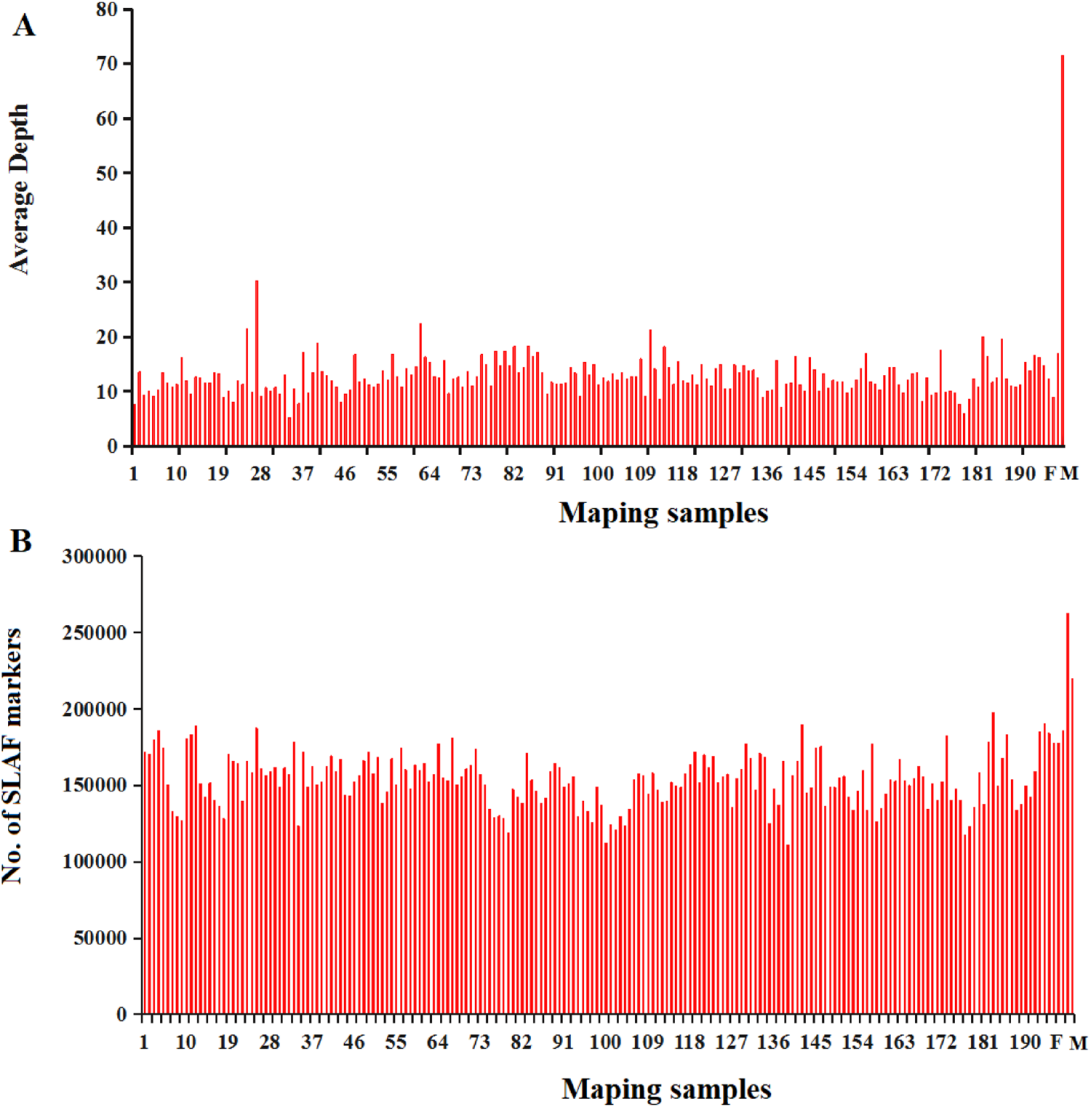
Average sequencing depth and number of markers for each of the F_2_ individual and their parents. The x-axis indicates the maping samples including female parent (F), male parent (M), and each of the F_2_ individuals; the y-axis indicates the average sequencing depth (**A**) and the number of SLAF markers (**B**).

All sequences were categorized into SLAF clusters based on similarity. Using high-throughput sequencing to eliminate the sequences with low lengths, repeats, and unconfirmed SLAFs, the residual, valid SLAF markers obtained were 262,222 and 219,948 in the male and female parents, respectively. The read numbers for SLAFs were 18,747,434 and 13,716,859 in the male and female parents, respectively; with average development rates of 71.50- and 62.37-fold, respectively. Through high-throughput sequencing, the SLAF markers recognized in the F_2_ were 110,940–197,542, with an average of 153,652. The SLAF markers developed ranged from 639,392 to 4,736,985 with an average of 1,969,377; the average development rates were 5.19- and 30.27-fold with a total average coverage rate of 12.82/individual (Fig. 1).

**Figure 2:**
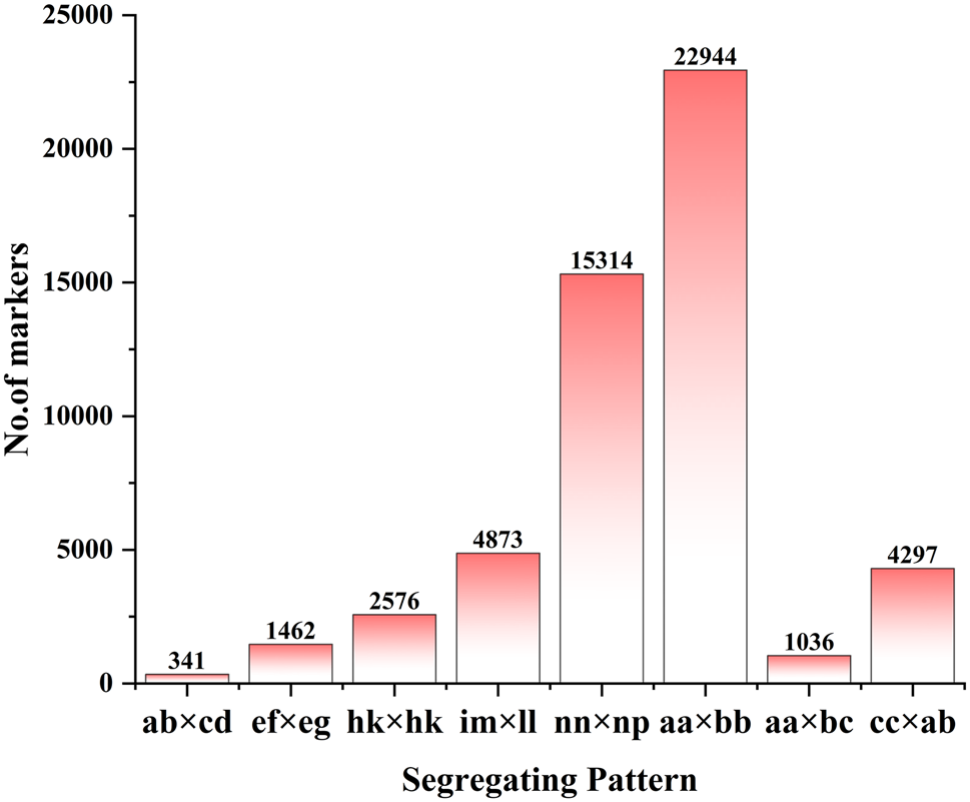
Number of polymorphic SLAF markers for eight segregation patterns. The x-axis indicates eight segregation patterns of polymorphic SLAF markers; the y-axis indicates the number of markers.

Based on the polymorphic differences between alleles and genes, 302,295 high-quality SLAF markers were developed, which consisted of three types: polymorphic, non-polymorphic, and repetitive polymorphic slotted markers (Table 1). Among them, 79,364 (26.25%) were polymorphic, which were subsequently used for genotyping. After removing low-quality polymorphic markers such as missing parental information, areas with repeat sequences, low integrity non-polymorphic markers (73.08%), and repeat polymorphic markers (0.67%), the remaining 53,023 high-quality and applicable SLAF markers were classified into eight segregation patterns, which were ab × cd, ef × eg, hk × hk, lm × ll, nn × np, aa × bb, ab × cc, and cc × ab (Fig. 2). An F_2_ population is obtained by selfing the F_1_ of a cross between two fully homozygous parents with genotype aa or bb. Therefore, only aa × bb isolates were used for genetic map construction, and 22,944 SLAF markers were classified into this genetic group. Of them, 3952 originated from the parents with an average sequencing depth of 66.925-fold, which in the F_2_ was 12.82-fold. All these SLAF markers were used to construct the genetic maps.

**Table 1:** SLAF mining results.

Type	Number of SLAF markers	Number of reads	Ratio (%)
Polymorphism	79,364	110,270,167	26.25
Non-polymorphism	220,904	311,617,085	73.08
Repetitive	2027	2,895,714	0.67
Total	302,295	422,400,985	100

### Routine characteristics of the genetic map

After a series of screenings, 3605 practical SLAF markers were obtained from a total of 3646 and used for the final linkage analysis. The residual 41 could not be mapped to any of the linkage maps. These markers had coverages of 165.30-fold and 98.59-fold in the male and female parents, respectively, and 20.14-fold in the F_2_ offspring. In these, the integrity of each marker was the critical parameter in controlling the quality of the genetic map. The average integrity of the mapped markers was 98.95%, indicating a relatively high quality of the genetic map.

**Figure 3:**
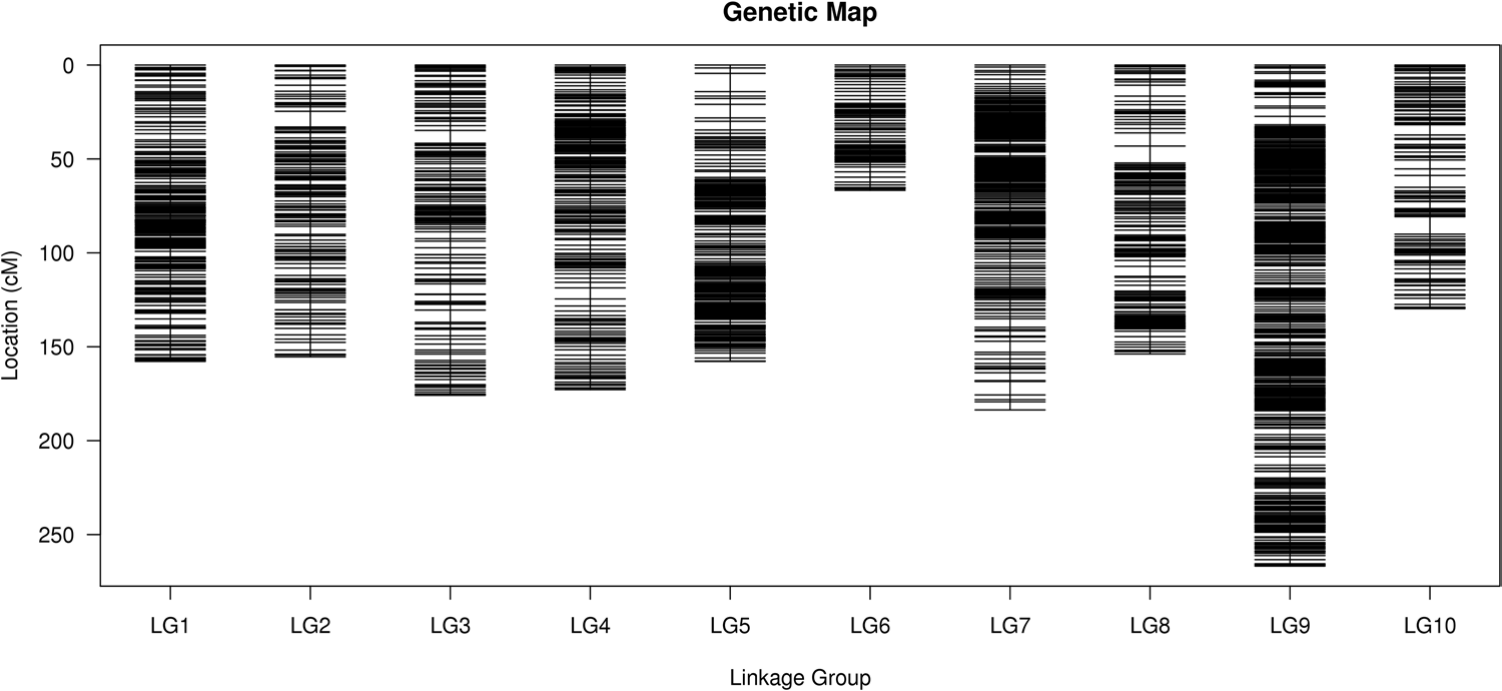
Distribution of SLAF markers on the 10 linkage groups of *C. lacryma-jobi*. A black bar indicates a SLAF marker. A red bar indicates a segregation distortion marker. The x-axis represents the linkage group number and the y-axis indicates the genetic distance (cM) in each linkage group.

For genetic mapping, 3952 SLAF markers from the 200 offspring were used. Linkage analysis was also achieved through Mendelian inheritance ratios by employing the JoinMap 4.1 software (https://www.kyazma.nl/index.php/JoinMap). Under conditions of LOD ≥ 4.0 and a recombination rate (r) ≤ 0.30, 3605 (98.88%) SLAF markers of 3646 were distributed across ten linkage groups (LGs), with a total map length of 1620.39 cM and an average of 0.45 cM, which is the densest genetic linkage map in Job’s tears thus far. The marker distribution and lengths of the LGs were not the same (Table 2, Fig. 3 and Supplementary Material Presentation 1). The largest LG was LG9, with 774 markers, a total length of 266.78 cM, and an average intermarker distance of 0.35 cM. The smallest was LG6, with only 97 markers, a total length of 66.73 cM, and an average intermarker distance of only 0.70 cM. In all the ten LGs, the average number of SLAF markers was 360.5, the length ranged from 66.73 cM (LG6) to 266.78 cM (LG9), the intermarker distances were between 0.35 cM (LG9) and 0.84 cM (LG10), the linkage levels were 98.06–100% with an average of 99.42% under the conditions of ‘Gap ≤ 5’, and the highest interval length was 9.72 cM (LG5) (Table S2). In the above LGs, ten segregation distortion regions (SDRs) were identified that accounted for 0.28% of the total SLAF markers, of which eight deviated in the male and two in the female parent; eight of them were distributed in LG7, and the remaining two in LG9.

**Table 2:** Distribution of SNP loci on the 10 linkage groups of *C. lacryma-jobi*.

LG ID	Marker number	Total distance (cM)	Average distance (cM)	Gaps ≤ 5(%)^a^	Max Gap	SDR
LG1	428	157.79	0.37	100	3.71	0
LG2	229	155.38	0.68	99.56	8.36	0
LG3	336	175.84	0.52	99.1	6.82	0
LG4	412	172.84	0.42	99.76	5.87	0
LG5	411	157.82	0.38	99.51	9.72	0
LG6	97	66.73	0.7	100	2.83	0
LG7	459	183.62	0.4	99.56	7.15	8
LG8	303	153.86	0.51	98.68	9.02	0
LG9	774	266.78	0.35	100	4.67	2
LG10	156	129.73	0.84	98.06	9.32	0
Maximum	774	266.78	0.84	100	9.72	8
Minimum	97	66.73	0.35	98.06	3.71	0
Total	3605	1,620.39	–	–	–	10
Average	360.5	162.04	0.45	99.42	9.72	–

^a^ 'Gap ≤ 5' indicated the percentages of gaps in which the distance between adjacent markers was smaller than 5 cM.

### Distribution of markers on the genetic map

Of the total 5378 SNP markers, 1519 SNP switch and 3859 SNP conversion points were recognized in the genetic map constructed with 3605 SLAF markers; the ratio between the switch and conversion points was 0.39. An analysis of the distribution rule of the markers for each LG revealed that the highest number of SNP markers was 1382 in LG9; 372 of them belonged to switch points, and the rest 1010 to conversion points (Table 3). The ratio of the switch and conversion points was 0.37. LG6 had the smallest number of 147 SNP markers; 54 of them belonged to switch points, and the rest 93 to conversion points; the ratio was 0.58 (Table 3). In total, 5387 SNP loci were detected among the 3605 SLAF markers on the final map (Table 3). Further analysis of these SNPs showed the transition type in a majority of them; it reached 71.76%, including the R (A/G) and Y (C/T) types, with 35.35% and 36.41%, respectively. The transversion type was lesser at 28.4%, including M (A/C), W (A/T), S (C/G), and K (G/T) types, among which the ratios ranged from 6.66 to 7.49%, respectively (Table 3).

**Table 3:** Statistics of mapped SNP marker types.

Linkage group	SNP number	Transition/Transversion number
LG1	541	388/153
LG2	268	194/74
LG3	440	320/120
LG4	623	452/171
LG5	613	418/195
LG6	147	93/54
LG7	755	545/210
LG8	428	308/120
LG9	1382	1010/372
LG10	181	131/50

### Quality evaluation of the genetic map

The quality of the genetic map was evaluated through the construction of haploid and thermal maps. The haplotype maps, which reflect the double exchange of the population, were developed for the parental controls and 200 offsprings using 3605 SLAF markers (Supplementary Material Presentation 2). Most recombinant areas could be clearly determined by their color differences, which indicated the occurrence of reorganization events. The missing markers for each LG ranged from 0.05% (LG4) to 1.9% (LG7). Most LGs were evenly distributed, indicating that the genetic map had a very high quality (Table 4).

**Table 4:** Statistics of mapped SNP marker types.

SNP types	Number	Ratio (%)
R(G/A)	1901	35.35
Y(T/C)	1958	36.41
S(G/C)	392	7.29
W(A/T)	403	7.49
K(G/T)	358	6.66
M(A/C)	366	6.81
Total	5378	100

A genetic map is a fundamental multi-point recombination analysis in which a lower recombination rate indicates a closer intermarker distance. The potential layout problems could be identified through an examination of the recombination relationships between each marker and the surrounding ones. The recombination relationship between LG markers can be demonstrated by a thermography map, which was constructed from multiple comparisons of the recombination levels among the 3,605 SLAF markers (Supplementary Material Presentation 3). The linkage relationship between adjacent markers on each LG enhanced with an increase in the distance, while that between a marker and distant ones gradually weakened, indicating that their order was correct.

### Segregation distorted markers on the map

The Chi-square test showed that 593 of the 3605 SLAF markers present on the genetic map were distortion markers, which reached 16.45% at P < 0.05. They were distributed on all ten LGs with varying proportions; their distribution mode in each LG was similar to that of all markers, except in LG7, 8, and 10 (Table 5). LG10 had the highest proportion of these markers at 33.33%, while LG3 had the lowest at 1.79%. The largest LG, LG9, had 101 distortion markers with a distortion ratio of 13.05%. In LG8 and 10, their frequency was remarkably higher than the other LGs, which were 32.67% and 33.33%, respectively.

**Table 5:** Distribution of segregation distorted markers.

Linkage group	All marker		Segregation distortion marker		χ^2^	*P*	Frequency of segregation distortion marker (%)	SDR number
	Number	Percentage (%)	Number	Percentage (%)				
1	428	11.87	88	14.84	0.465	0.203	20.56	0
2	229	6.35	20	3.37	0.865	0.862	8.73	0
3	336	9.32	6	1.01	0.008	0.158	1.79	0
4	412	11.43	68	11.47	1.213	0.032	16.50	0
5	411	11.40	17	2.87	0.043	0.313	4.14	0
6	97	2.69	15	2.53	0.527	0.005	15.46	0
7	459	12.73	127	21.42	0.628	0.014	27.67	8
8	303	8.40	99	16.69	0.802	0.307	32.67	0
9	774	21.47	101	17.03	0.032	0.504	13.05	2
10	156	4.33	52	8.77	0.824	0.014	33.33	0
Total	3605		593				17.39	10

χ^2^ and *P* indicate c2 values with one degree of freedom and the corresponding probability, respectively. SDR means segregation distortion region.

## Discussion

Developing abundant and reliable molecular markers is of great significance for genetic map construction. SNP markers have been widely used for this purpose. Compared to this common method, SLAF-seq technology used for large-scale marker development provided a higher density, better consistency, improved effectiveness, and saved costs^19^. It has been utilized for the development of molecular markers in a number of plants and genetic mapping in laurel^20^, mango^18^, walnut^21^, soybeans^17^, and other essential crops. The rapid development of this technology proved its effectiveness for the large-scale development of molecular markers and high-density genetic mapping.

This study used the SLAF reduced-representation genome sequencing method for large-scale marker development in Job’s tears. The high-throughput sequencing of the SLAF library used an Illumina^®^ sequencing platform, producing 325.49 GB of raw data consisting of 1,628,398,591 paired-end sequencing fragments. A total of 302,295 SLAF markers were obtained through comparative and clustering analysis, of which 3605 practical, highly polymorphic markers were identified after filtering and eliminating the low-quality markers and were used for the linkage map construction. In this study, numerous molecular markers were determined to provide a robust foundation for genetic map construction. Abundant genome information provides an essential reference for further molecular biology investigations in Job’s tears.

A crucial advantage of SLAF-seq is the capacity to develop large-scale molecular markers in a single experiment. However, the data obtained by high-throughput sequencing inevitably included missing numericals and errors. Therefore, multiple selections must be used to eliminate these errors, which otherwise can affect genetic map construction^19^. This study obtained 79,364 polymorphic SLAF markers from the raw sequence data. However, only 3605 useful SALF markers were finally identified after eliminating those markers rendered ineffective, possibly due to missing parental genotype, low integrity, Mendelian errors, and significant segregation. In addition, the sequencing results indicated that the accuracy of SLAF can be effectively improved by enhancing the sequencing depth, which was consistent with previous studies^19,22^. Therefore, it is necessary to intensify the sequencing depth in future experiments for efficient marker development.

The GC content of the SLAF library constructed in this study was 46.96%, which was slightly lower than the transcription group^23^, which might be due to different origins of the DNA sequence, i.e., genomic, cDNA, or EST. The SNP sites in these SLAF markers mostly belonged to the conversion type (71.76%), which was similar to mangoes, peonies, and sesame seeds^16^. The polymorphism rate of the SLAF markers identified in the parents of the target group was 26.25%, which was lower than the EST-SSR polymorphism rate of 31.1% obtained previously^23^. However, this rate was higher than those of many other varieties of Job’s tears^11,12^, indicating the construction of a map with high genetic diversity among varieties through the use of SLAFs, which was consistent with previous investigations^2,24^.

In comparison with the genomes of the other annual homozygous herbs, the heterozygosity of the genome of Job’s tears was far more complex to be used for genetic map construction. Therefore, the marker segregation type of Job’s tears was much more complex and quite different from other traditional segregation populations. In this study, ‘Wenyi No. 2’ and ‘Qianyi No. 2’ were used as male and female parents, respectively, and the F_2_ individuals were used for genetic mapping. Besides, the highly heterozygous and more complex genome of Job’s tears, with a size of 1.6 to 1.73 GB, makes it harder to construct high-density genetic maps^12,25^. Therefore, it is imperative to find a proper method for high-density linkage map construction in Job’s tears.

A two-step method that optimizes the construction of high-density linkage maps through the use of the K-nearest neighbor and maximum likelihood algorithms was also applied in Job’s tears by employing repeated sorting and error correction strategies^26^. Compared with the traditional JoinMap software, this method was more efficient for high-throughput sequencing, more accurate and reliable for linkage map construction, and more effective for mapping. This mapping strategy has been widely used in mangoes^18^, laurels^20^, walnuts^21^, and peonies^27^, and its applicability was confirmed by this study in Job’s tears.

Genetic map is the basis of QTL mapping, map-based cloning, molecular marker-assisted breeding and comparative genome research for quantitative traits. Therefore, the construction of a high-density genetic map for the study of Job’s tears traits is of great significance, but the study of C. lacryma-jobi genetic map started late. Qin et al. the first genetic map of *C. lacryma-jobi* was constructed by using AFLP and RFLP markers and the strategy of 'quasi-cross test'^11^. However, due to the difficulties in population construction and the limited number of markers, the study on genetic map of *C. lacryma-jobi* is very slow. Guo et al. constructed an F2 population (551 individual plants) of *C. lacryma-jobi*, consisting of 'Daheishan' (Male parent) and 'Xiaobaike' (Female parent). The map contained 230 InDel markers, with a length of 1570.12 cM and an average spacing of 6.83 cM between the markers. The genes *Ccph1* and *Ccph2* for seed coat thickness and color were accurately identified^12^. Compared with previous studies, although this map has shown a significant improvement in marker density, there were multiple large gaps between adjacent markers on the map, which made it difficult to widely apply the constructed map to QTL localization analysis. This study built the first high-density genetic map of Job’s tears, in which 3605 SLAF markers were clustered into ten LGs obtained within the same ploidy (2n = 20) as the Beijing Job’s tears species^28^. The total length of the map was 1620.39 cM, with an average intergenic distance of 0.45 cM. Each LG had an average of 360.5 markers, which varied widely, with some clustering predominantly in particular regions, especially in LG1, 7, and 9. This phenomenon might be caused by factors such as inconsistent molecular marker polymorphism or inconsistent recombination rates in certain regions of the chromosomes in graph-based parents^19^. Similar phenomena were also observed in plants such as peonies^27^ and sunflowers^29^. The clustered aggregation of sequences in the LG was considered to be related to the chromosomal filament or hesochtine region^30^. The genetic map constructed using this method has the highest density and the highest number of markers, far exceeding the reported genetic map.The ideal linkage group should be consistent with the chromosomes of the species, but there are still gaps in some positions on the map, indicating that the map is not fully saturated. In future research, increasing the number of mapping populations and markers, it is expected to solve these problems and further improve the saturation and density of the genetic map.In this study, the first high-density genetic linkage map of *C. lachryma-jobi* was constructed, which will lay an important foundation for QTL analysis, gene map cloning and molecular marker-assisted selection breeding of important traits of *C. lachryma-jobi*.

Segregation distortion (SD), even though it is widespread throughout nature and is considered to be one of the crucial drivers of species evolution, the underlying causes are still doubtful and controversial^31^. It may be due to biological factors, including the selection of gametes and zygotes, non-homogenous recombination, translocation on chromosomes or non-homogenous sites, and low homozygosity of the parents used for mapping^32^. Other reasons might be environmental factors, experimental errors, offspring separation, and the loss of chromosomes^27,33–35^. In this study, ~ 16.45% of the 593 markers showed significant (P < 0.05) segregation distortion in the target group, indicating that the genetic diversity of the parents was high. All 593 markers formed ten SDRs and were distributed in the LGs as clusters. The high incidence of SD might be based on the gametophyte and sporophyte selected^36^ and is widely found in plants^13,16,35^. In addition, the use of such markers to build a linkage map can enhance its coverage^16,33^ and may be conducive to QTL positioning^37,38^.

This study constructed the first high-density genetic linkage map, which provides an effective way for critical trait analysis through QTLs, map-based cloning, and MAS for breeding in Job’s tears. The 3605 SNP markers constituted ~ 93.78% of the total map and were the most common dominant gene sequence labels, which could help in comparative genomic research^39^ and association mapping^40^. More importantly, the SLAF markers utilized for building high-density genetic maps were developed at the genome-wide scale.

This study demonstrated the applicability of SLAF-seq technology for identifying large-scale genetic markers in Job’s tears. Additionally, HighMap was recognized as an effective tool for developing molecular markers and constructing high-density linkage maps by using high-throughput sequencing data. This work provides an essential molecular basis for examining genetic diversity, variety identification, location of phenotype-associated QTLs, and functional and structural genomics in Job’s tears.

## Materials and methods

### Plant materials and DNA extraction

The F2 mapping population from a Cross between ‘Wenyi No. 2’ (female parent) and ‘Qianyi No. 2’ (male parent) consisted of 200 individuals grown at the Institute of Subtropical Crops of the Guizhou Academy of Agricultural Sciences, Guiyang, China. ‘Wenyi No. 2’ has red anthers, black shells, and red leaves. An essential trait of ‘Wenyi No. 2’ is its resistance to Smut, to which it is exposed frequently during its cultivation and postharvest storage, and severely affects the development of the Job’s tears industry. ‘Qianyi No. 2’ has yellow anthers, white shells, and green leaves. However, it is susceptible to Smut. The healthy leaves from the parents and F2 offspring were collected and stored in liquid N2. All methods were carried out according to the relevant guidelines and regulations. All experimental materials were obtained from the Guizhou Institute of Subtropical Crops, and the F2 generation seeds were planted at the field of the Guizhou Institute of Subtropical Crops. For DNA extraction, the modified CTAB buffer (8.18 g NaCl, 2 g CTAB, 20 mM EDTA, and 100 mM Tris [pH ± 8.0] with a final volume of 100 mL) and the traditional CTAB method were utilized^16^. The total genomic DNA of each plant was extracted and analyzed by electrophoresis with 1% agarose gel and quantified by a NanoDrop™ 2000 spectrophotometer (Thermo Fisher Scientific, MA, USA).

### Construction of an SLAF library and high-throughput sequencing

SLAF-seq method^13^ was used. First, the genome of the parents and F2 population was digested by Hpy166II (New England Biolabs, MA, USA). Then polyA as dATP was ligated to the end of the digested fragment by employing the Klenow fragment (3′–5′ exo-) (New England Biolabs) at 37 ℃. Next, the PAGE-purified dual-label sequencing markers (Life Technologies, CA, USA) were ligated to the newly added terminal polyA utilizing the T4-DNA ligase. PCR was performed with the diluted DNA samples, and the forward: 5′-AATGATACCGACCACCGA-3′ and reverse: 5′-CAAGCAGAAGACGGCATA-3′ primers, Q5^®^ High-Fidelity DNA Polymerase (NEB), and dNTPs. The PCR products were purified and collected by Agencourt AMPure XP beads (Beckman Coulter, High Wycombe, UK) and separated by electrophoresis on a 2% agar gel. The DNA fragment (with indices and adaptors) between 264 and 464 bp was electrophoresed again, and the band was extracted from the gel employing a QIAquick^®^ gel extraction kit (Qiagen, Hilden, Germany). The paired, terminal 125 bp sequences obtained were analyzed on a Hi-Seq 2500 system (Illumina Inc., CA, USA).

### SLAF-seq data and genotyping analyses

SLAF-seq data were analyzed, and genotyping was performed using a previously described method^13^. Dual index^41^ was selected for the identification of the raw sequencing data, and reads for each sample were used to evaluate their quality and quantity. SLAF labels were also developed in the parents and F_2_ individuals through read clustering. Polymorphism was analyzed based on the differences between the number of alleles and the gene sequences. The SLAF labels (polymorphic) with polymorphic sites (SNPs and InDels) were selected for subsequent analysis. The SLAF markers were evaluated and filtered multiple times to obtain high-quality and practically effective molecular markers. Since Job’s tears is diploid, the filtered DNA samples contained > four different suspect SLAF genotypes at one locus. In this study, the SLAF sequences with lengths < 200 bp were defined as short, filtered, and excluded. SLAFs with 2–4 tags were identified as polymorphic and considered potentially valuable tags. Polymorphic markers were divided into eight separation modes, which were ab × cd, ef × eg, hk × hk, lm × ll, nn × np, aa × bb, ab × cc, and cc × ab. The F_2_ population was obtained from genetically pure F_1_ parents with genotypes aa or bb. Therefore, the SLAF marker with the separation pattern aa × bb was used for genetic map construction (Table S2). Their average sequencing depths were > 66-fold in parents and > 12-fold in the progeny. Each progeny contained > 80% of these markers also detected in the parents, i.e., the SLAF markers in individuals had 80% integrity.

### Genetic map construction

LGs were initially divided based on the improved LOG score (MLOD) values ≤ 5 on the marker site. HighMap strategy was chosen for arranging the SLAF tags in a specific order and correcting the genotyping errors in the LGs to build more effective maps^26^. The genetic map was constructed by the maximum likelihood method^42^, and genotyping errors were corrected by the SMOOTH method^43^. The missing genotypes were estimated by the k-nearest neighbor algorithm^44^. The recombination rates between markers were calculated using the JoinMap 4.0 software. The r values were converted into genetic map distance expressed in cMs through the Kosanbi function^45^, and a high-density genetic chain map of Job’s tears was drawn. Areas with ≥ 3 partial separation markers in adjacent locations on the map were considered partial separation hot spots, which were defined as SDRs.

### Supplementary Information

Supplementary Information.

## Data Availability

The raw sequencing data and SLAL sequencing data generated in this study have been deposited to the National Genomics Data Center (NGDC, https://ngdc.cncb.ac.cn/) under project number PRJCA018829.

## Abbreviations

SLAF-seq: Specific-locus amplified fragment sequencing
cM: Centimorgan
QTL: Quantitative trait locus
SNP: Single nucleotide polymorphism
MAS: Marker-assisted selection
AFLP: Amplified fragment length polymorphism
RFLP: Restriction fragment length polymorphism
RAPD: Random amplified polymorphic DNA
ISSR: Inter-simple sequence repeat
SRAP: Sequence-related amplified polymorphism
SSR: Simple sequence repeats
LG: Linkage group
SDR: Segregation distortion region
SD: Segregation distortion
