# Supplementary figures and images for: Construction of a high-density genetic map based on large-scale marker development in Coix lacryma-jobi L. using specific-locus amplified fragment sequencing (slaf-seq)
Source: Sci Rep. 2024 Apr 26;14:9606. doi: 10.1038/s41598-024-58167-8 (PMC11053130; doi:10.1038/s41598-024-58167-8)

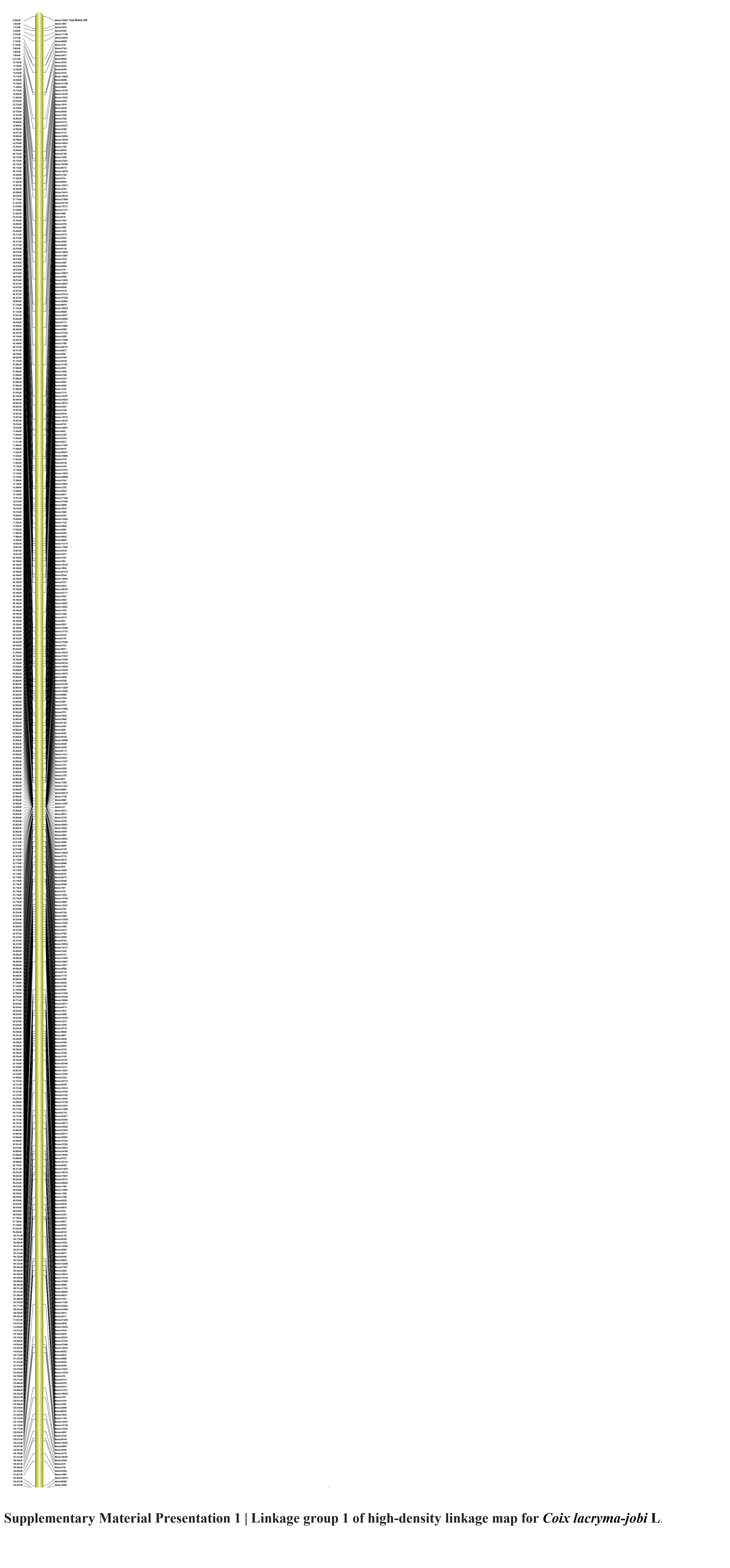

Supplement: Supplementary file 1 — Supplementary Information. [file 41598_2024_58167_MOESM1_ESM.zip › Supplementary material/Supplementary Material Presentation 1/LG1.genetic.png]

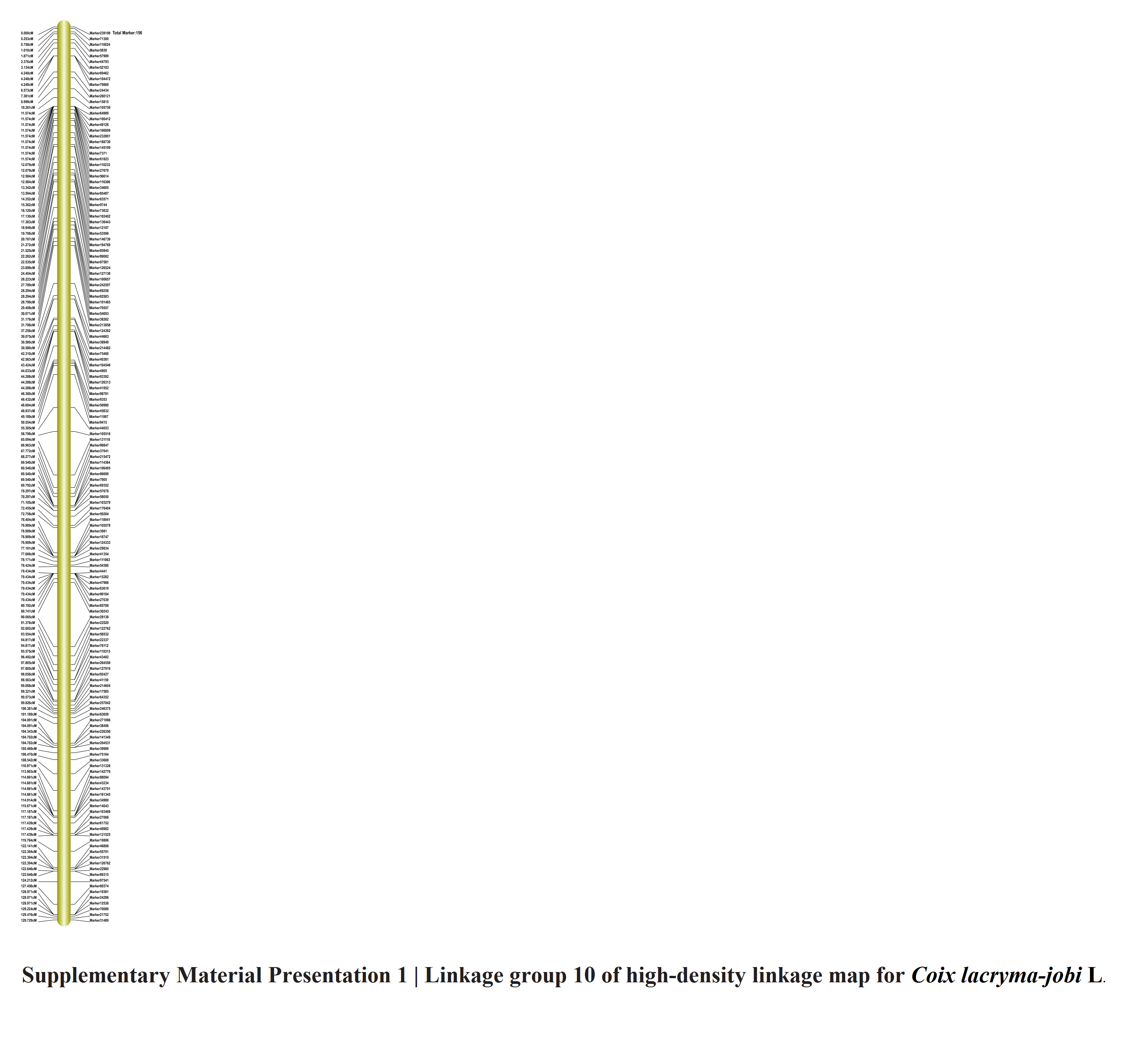

Supplement: Supplementary file 1 — Supplementary Information. [file 41598_2024_58167_MOESM1_ESM.zip › Supplementary material/Supplementary Material Presentation 1/LG10.genetic.png]

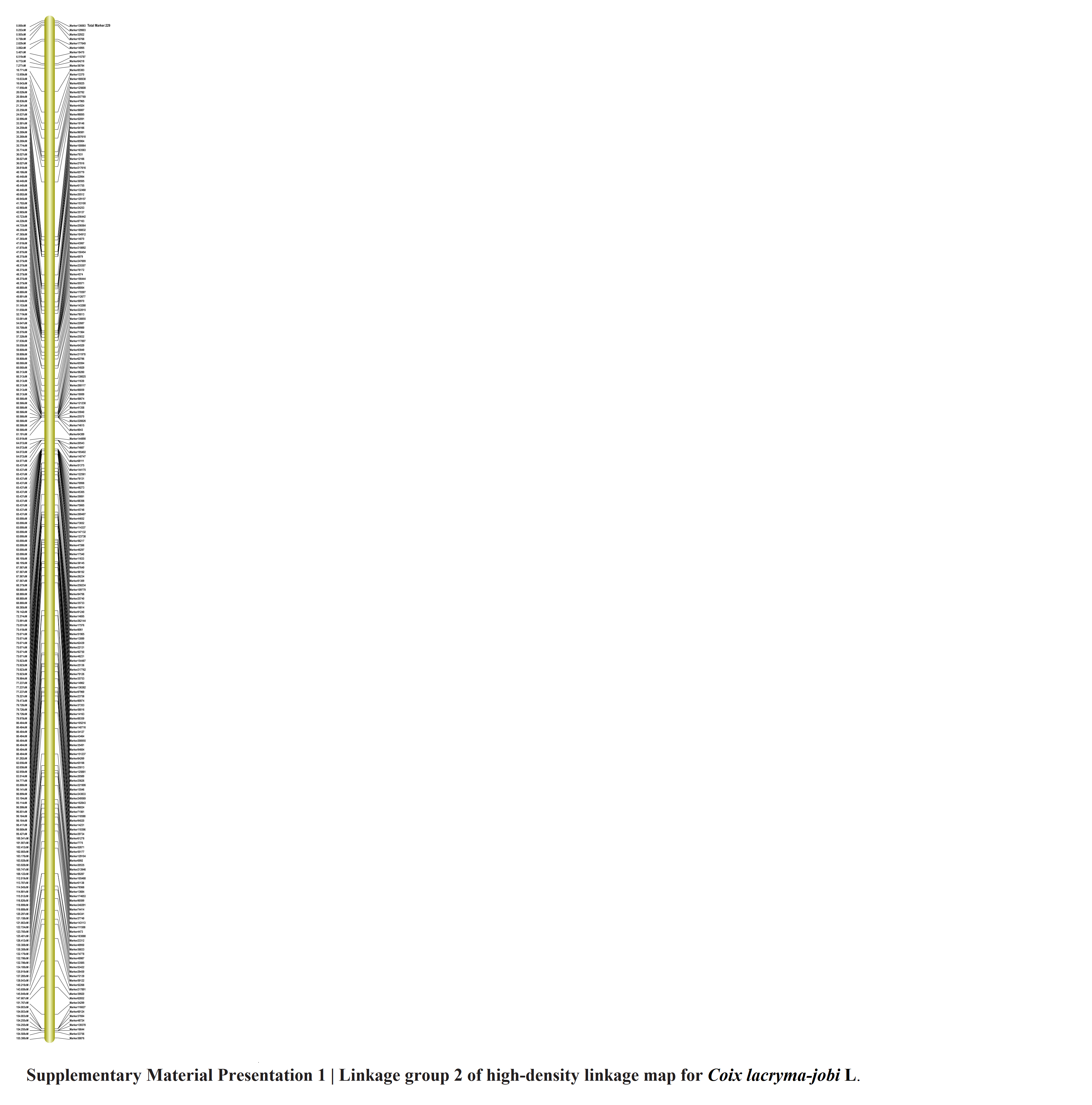

Supplement: Supplementary file 1 — Supplementary Information. [file 41598_2024_58167_MOESM1_ESM.zip › Supplementary material/Supplementary Material Presentation 1/LG2.genetic.png]

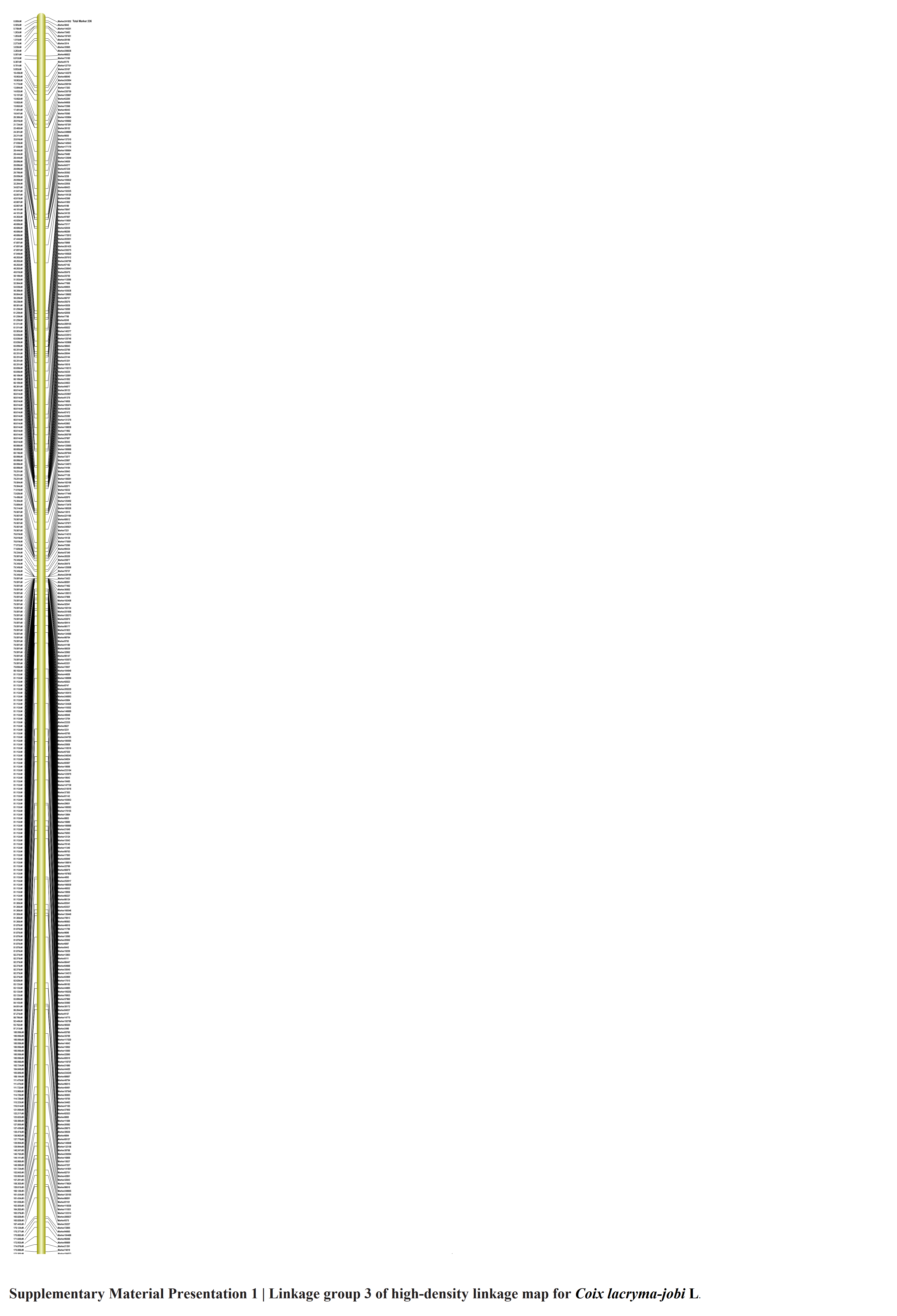

Supplement: Supplementary file 1 — Supplementary Information. [file 41598_2024_58167_MOESM1_ESM.zip › Supplementary material/Supplementary Material Presentation 1/LG3.genetic.png]

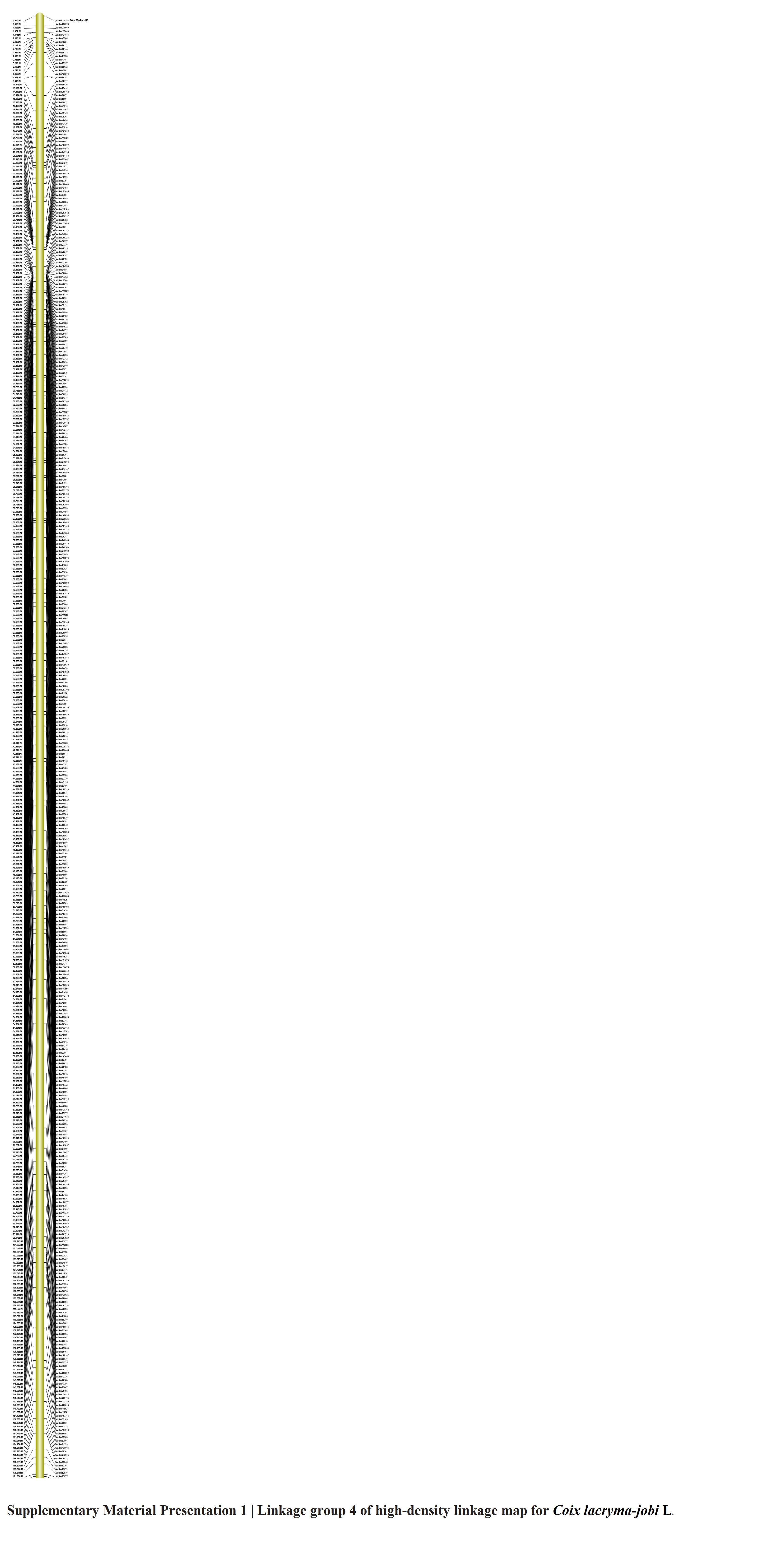

Supplement: Supplementary file 1 — Supplementary Information. [file 41598_2024_58167_MOESM1_ESM.zip › Supplementary material/Supplementary Material Presentation 1/LG4.genetic.png]

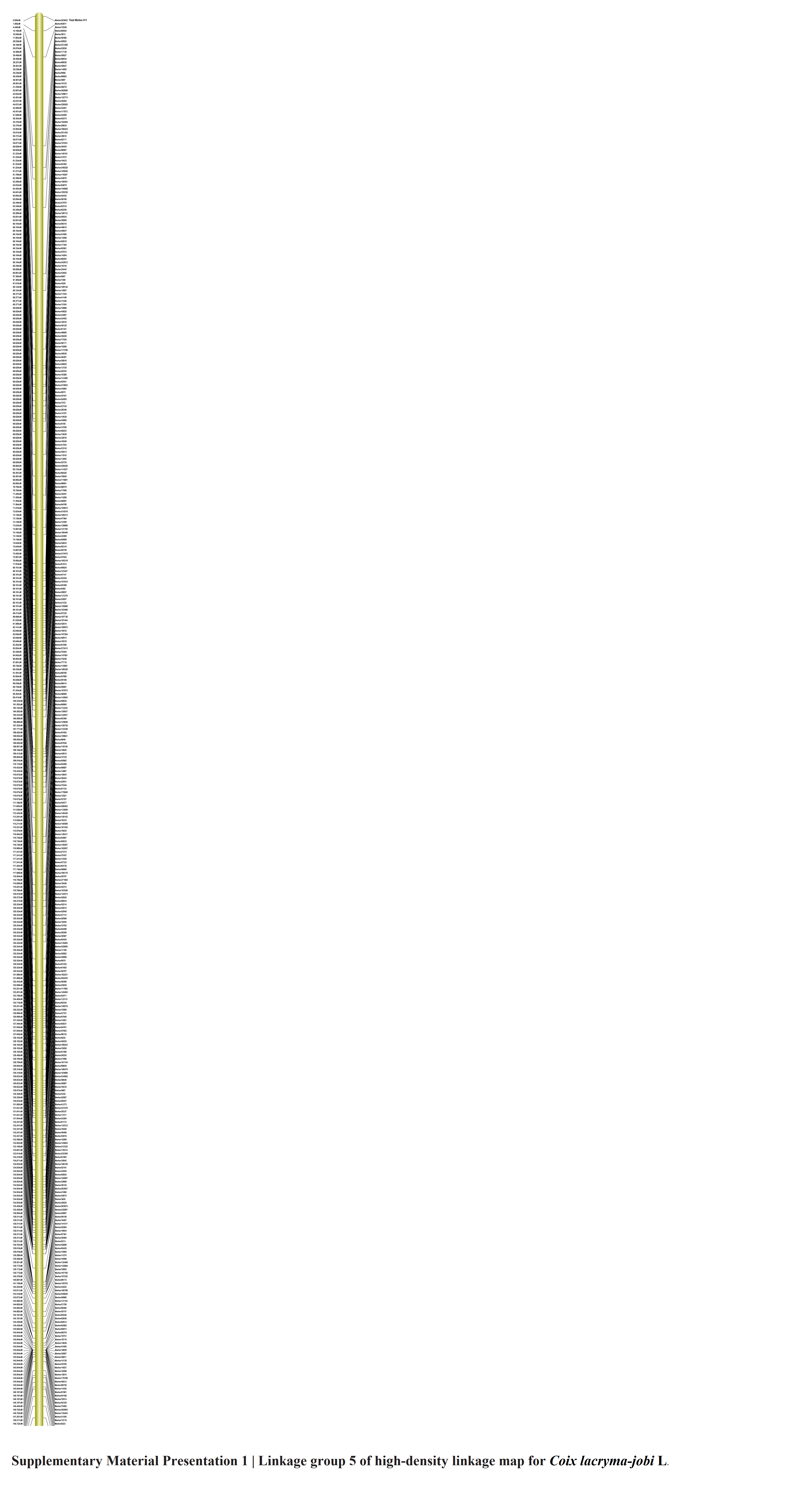

Supplement: Supplementary file 1 — Supplementary Information. [file 41598_2024_58167_MOESM1_ESM.zip › Supplementary material/Supplementary Material Presentation 1/LG5.genetic.png]

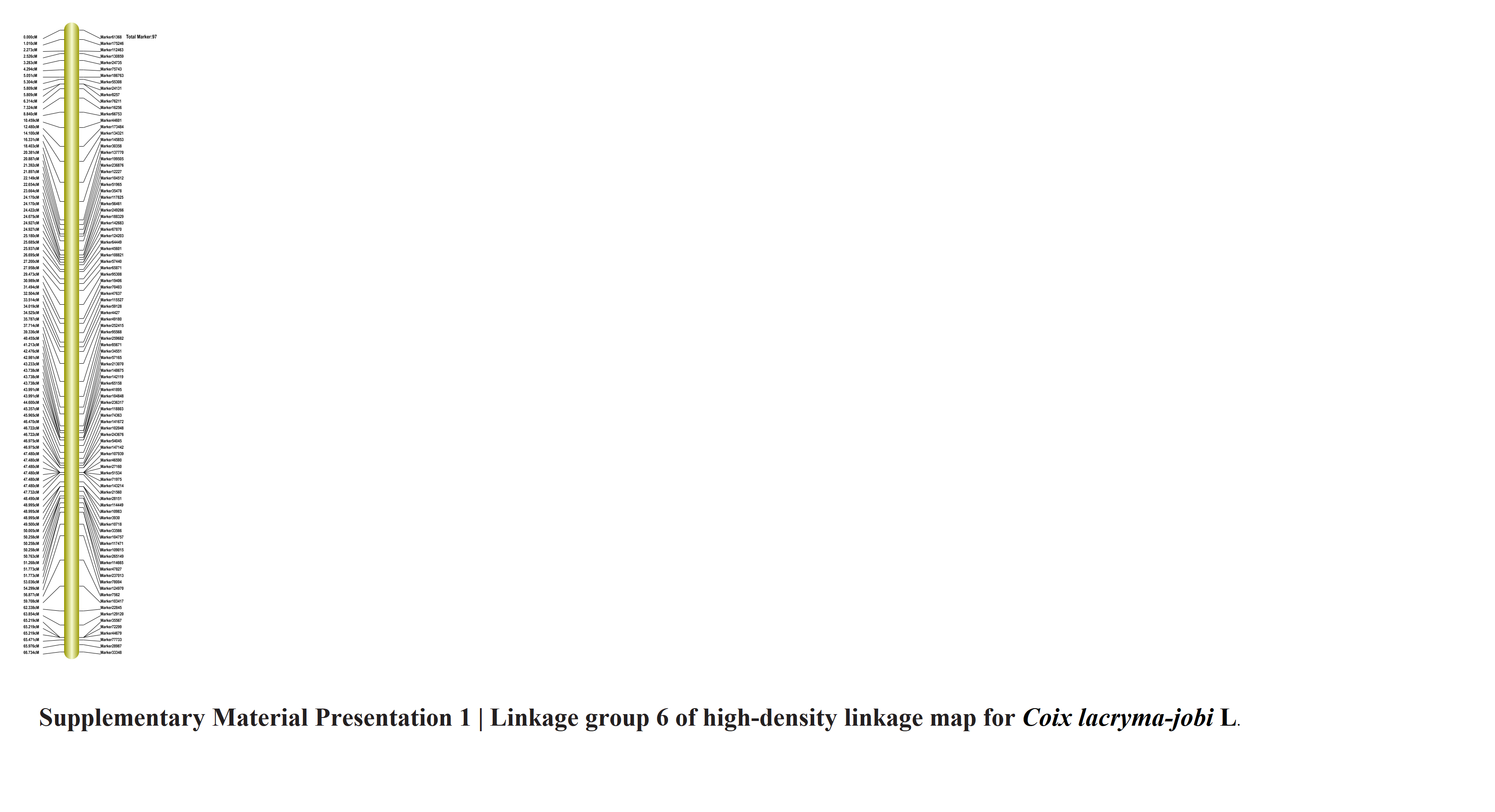

Supplement: Supplementary file 1 — Supplementary Information. [file 41598_2024_58167_MOESM1_ESM.zip › Supplementary material/Supplementary Material Presentation 1/LG6.genetic.png]

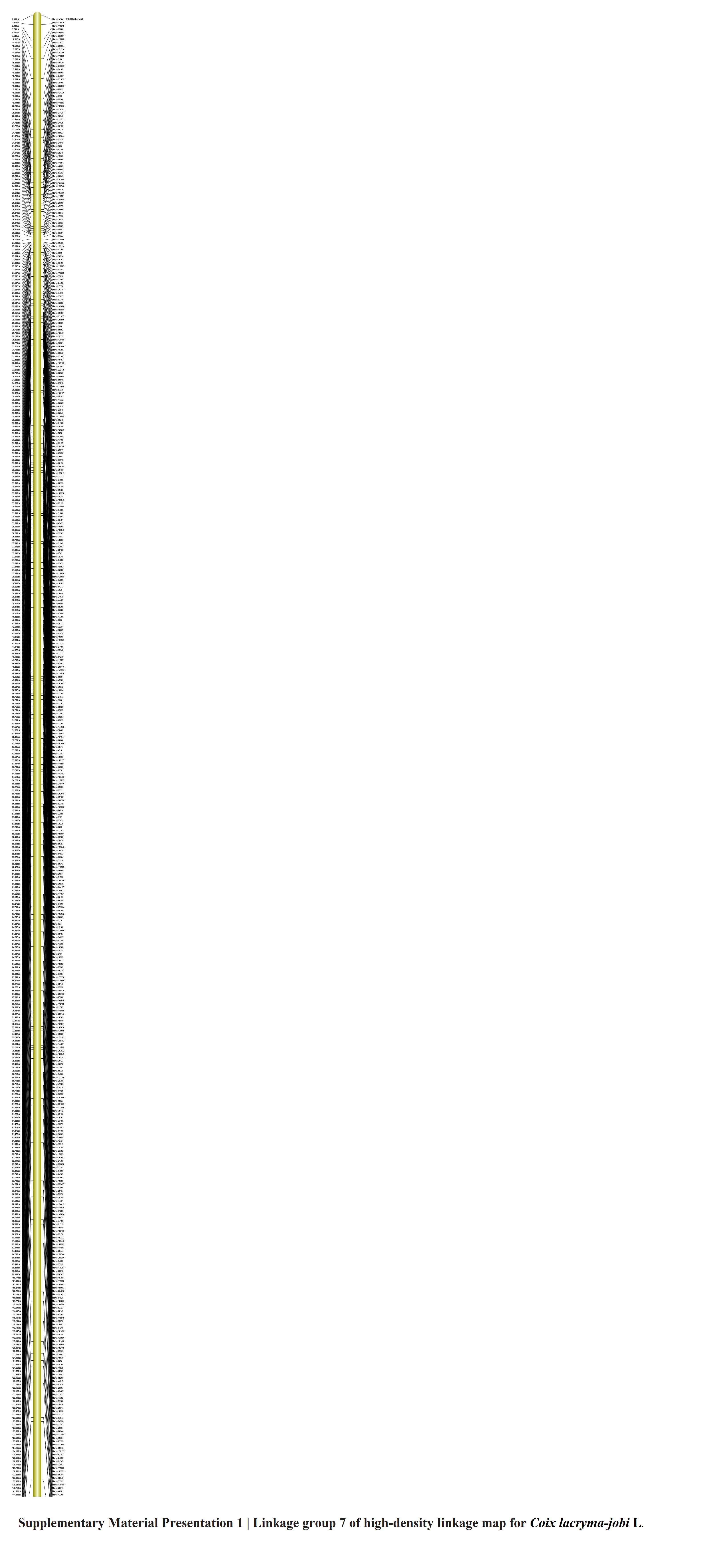

Supplement: Supplementary file 1 — Supplementary Information. [file 41598_2024_58167_MOESM1_ESM.zip › Supplementary material/Supplementary Material Presentation 1/LG7.genetic.png]

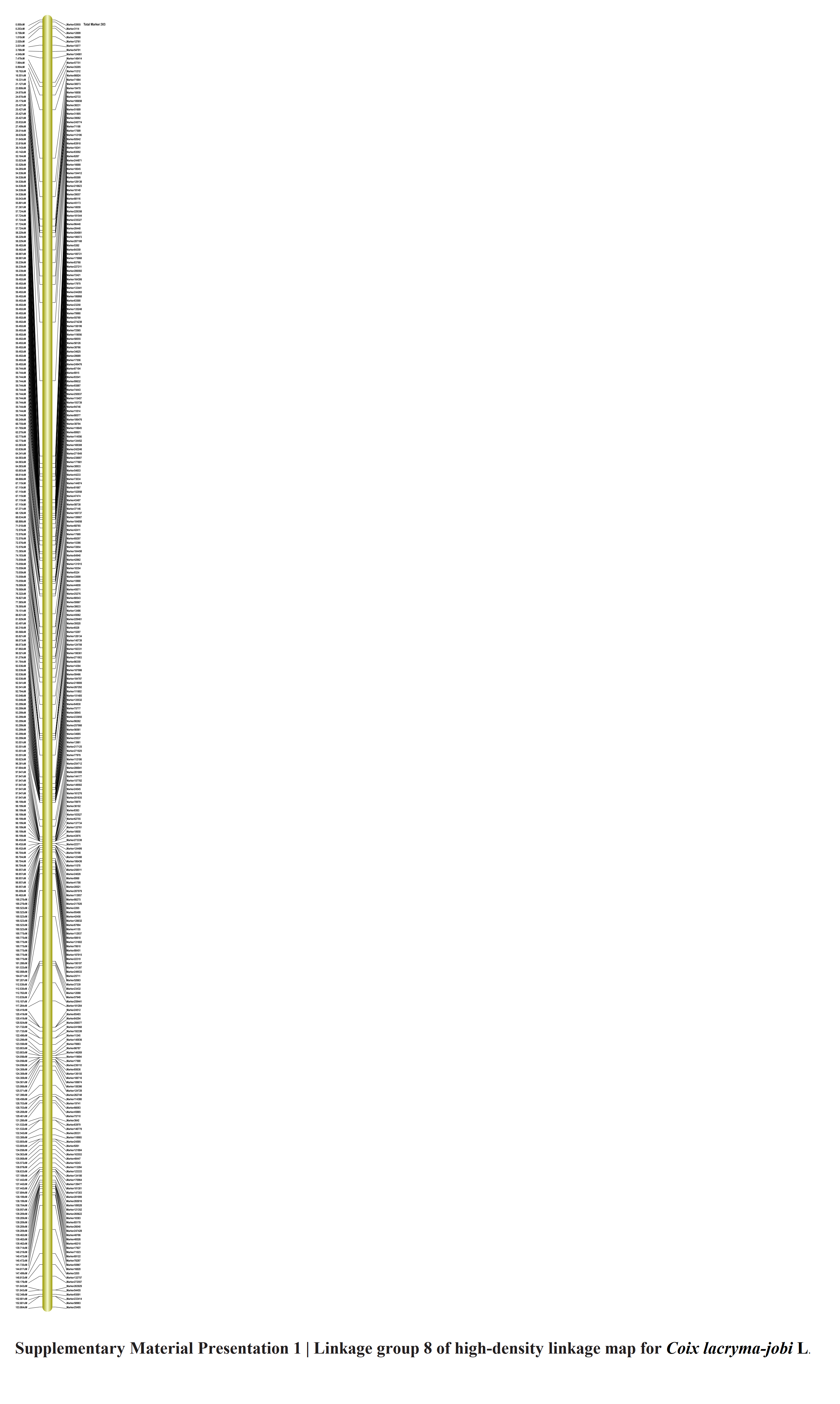

Supplement: Supplementary file 1 — Supplementary Information. [file 41598_2024_58167_MOESM1_ESM.zip › Supplementary material/Supplementary Material Presentation 1/LG8.genetic.png]

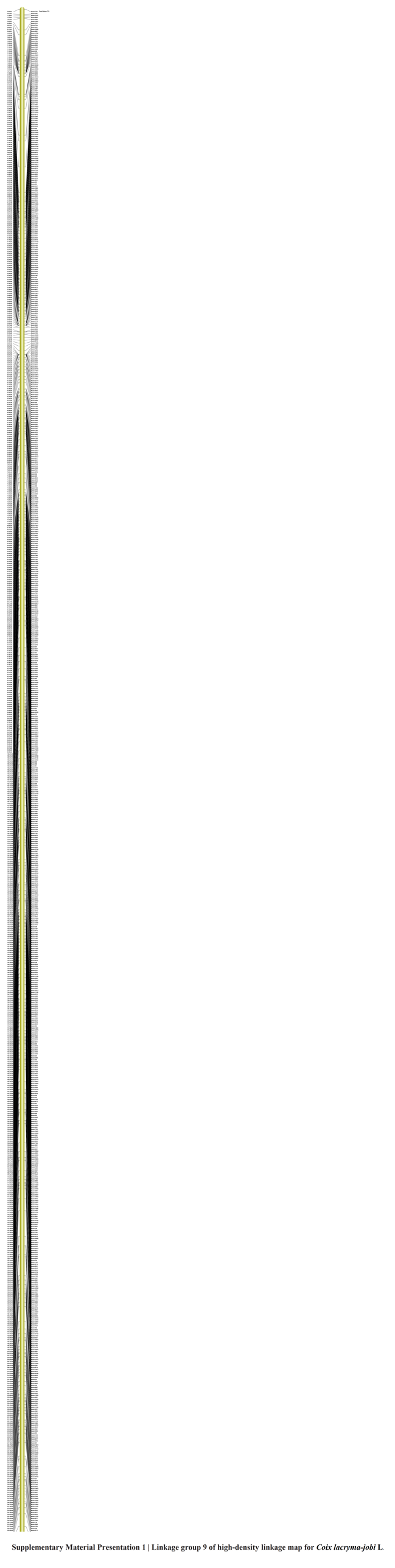

Supplement: Supplementary file 1 — Supplementary Information. [file 41598_2024_58167_MOESM1_ESM.zip › Supplementary material/Supplementary Material Presentation 1/LG9.genetic.png]

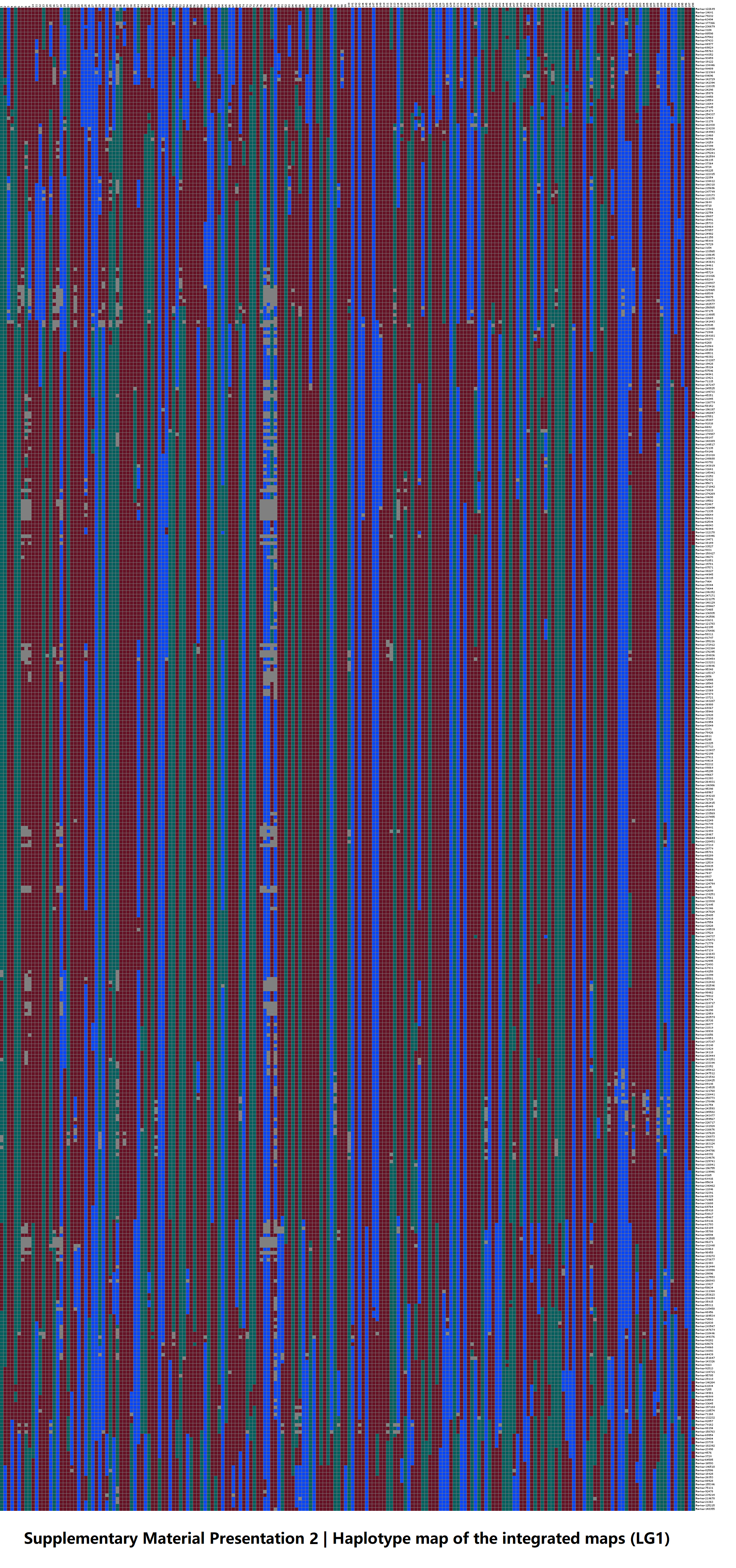

Supplement: Supplementary file 1 — Supplementary Information. [file 41598_2024_58167_MOESM1_ESM.zip › Supplementary material/Supplementary Material Presentation 2/LG1.haploMap.png]

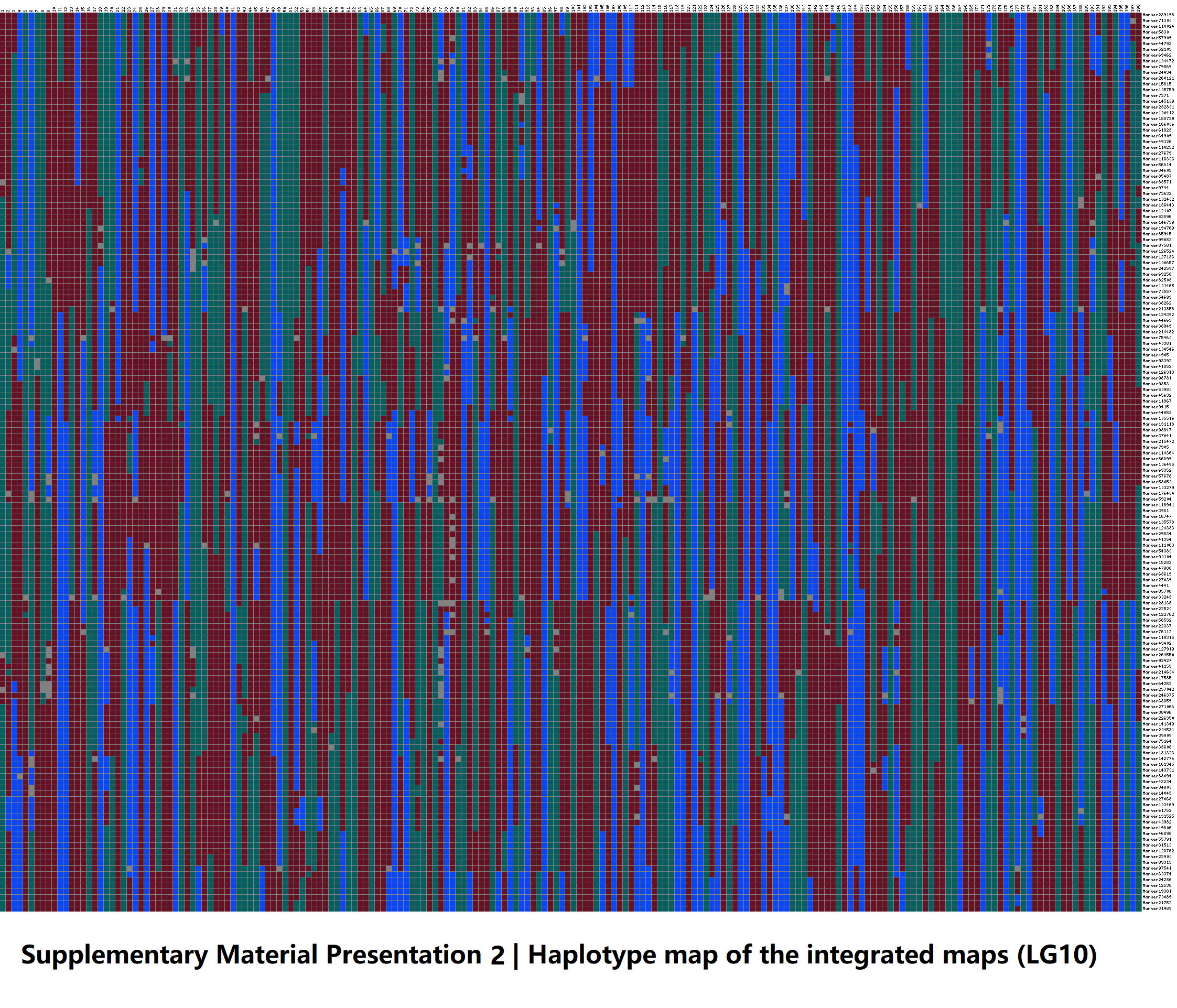

Supplement: Supplementary file 1 — Supplementary Information. [file 41598_2024_58167_MOESM1_ESM.zip › Supplementary material/Supplementary Material Presentation 2/LG10.haploMap.png]

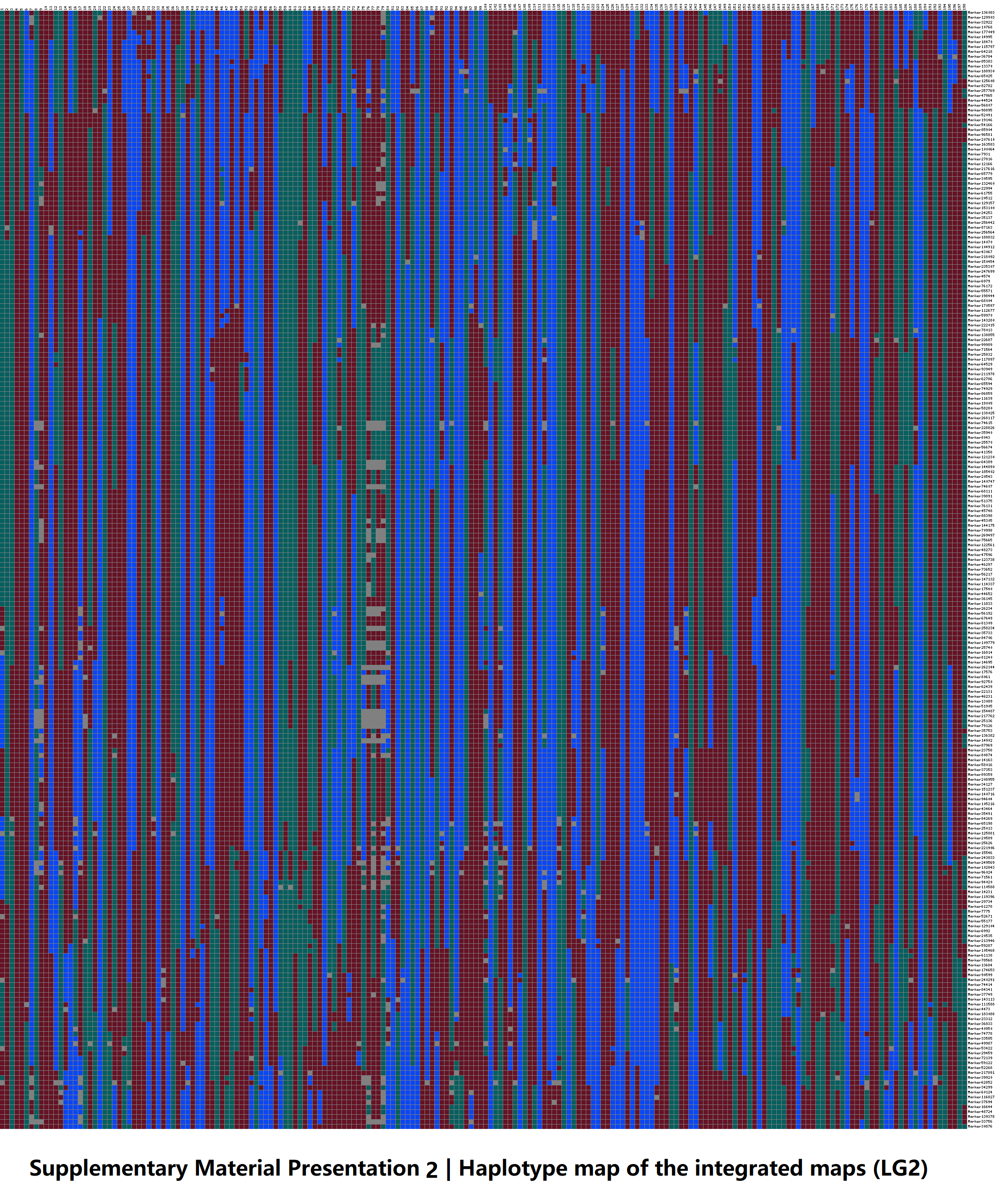

Supplement: Supplementary file 1 — Supplementary Information. [file 41598_2024_58167_MOESM1_ESM.zip › Supplementary material/Supplementary Material Presentation 2/LG2.haploMap.png]

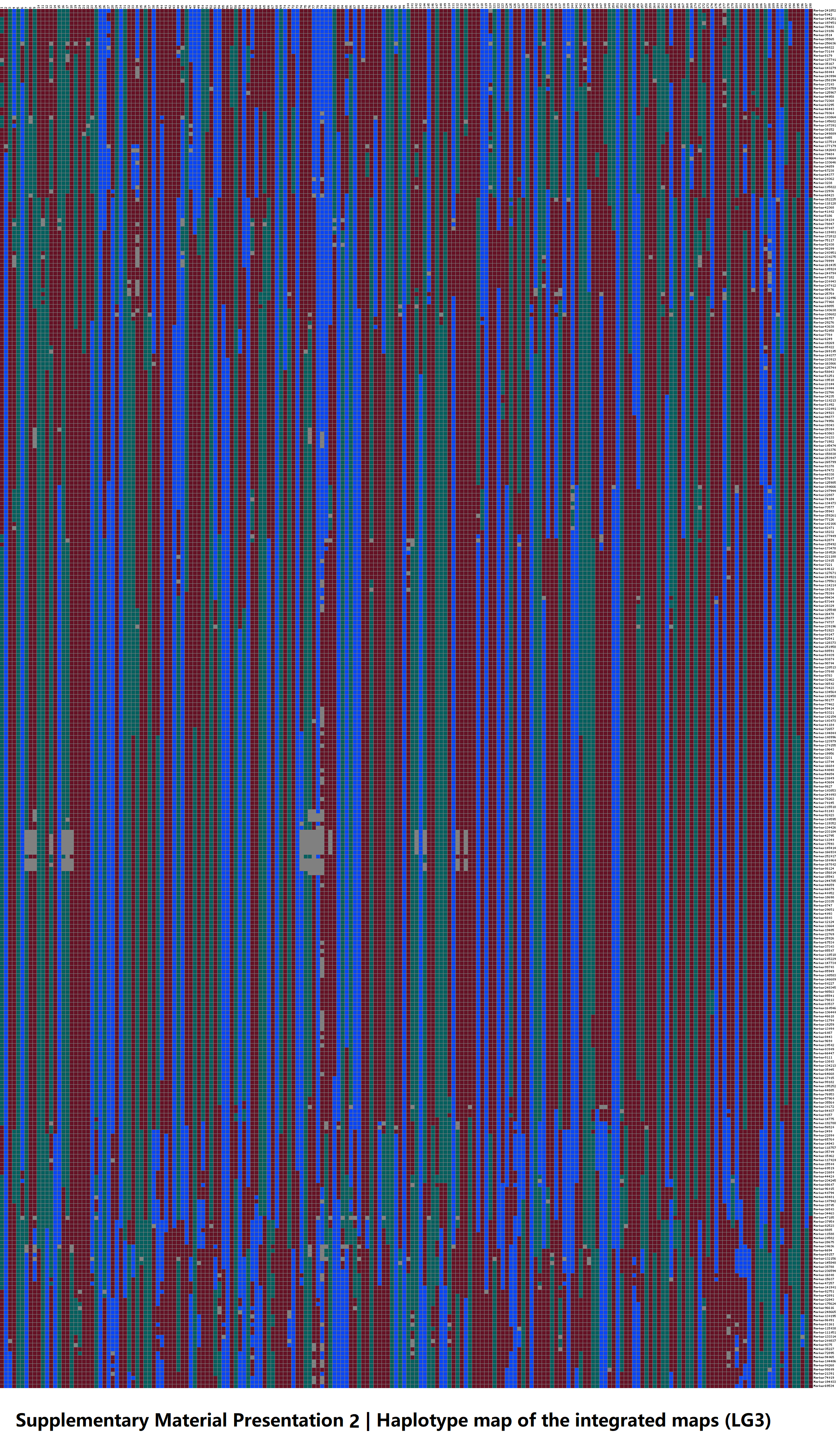

Supplement: Supplementary file 1 — Supplementary Information. [file 41598_2024_58167_MOESM1_ESM.zip › Supplementary material/Supplementary Material Presentation 2/LG3.haploMap.png]

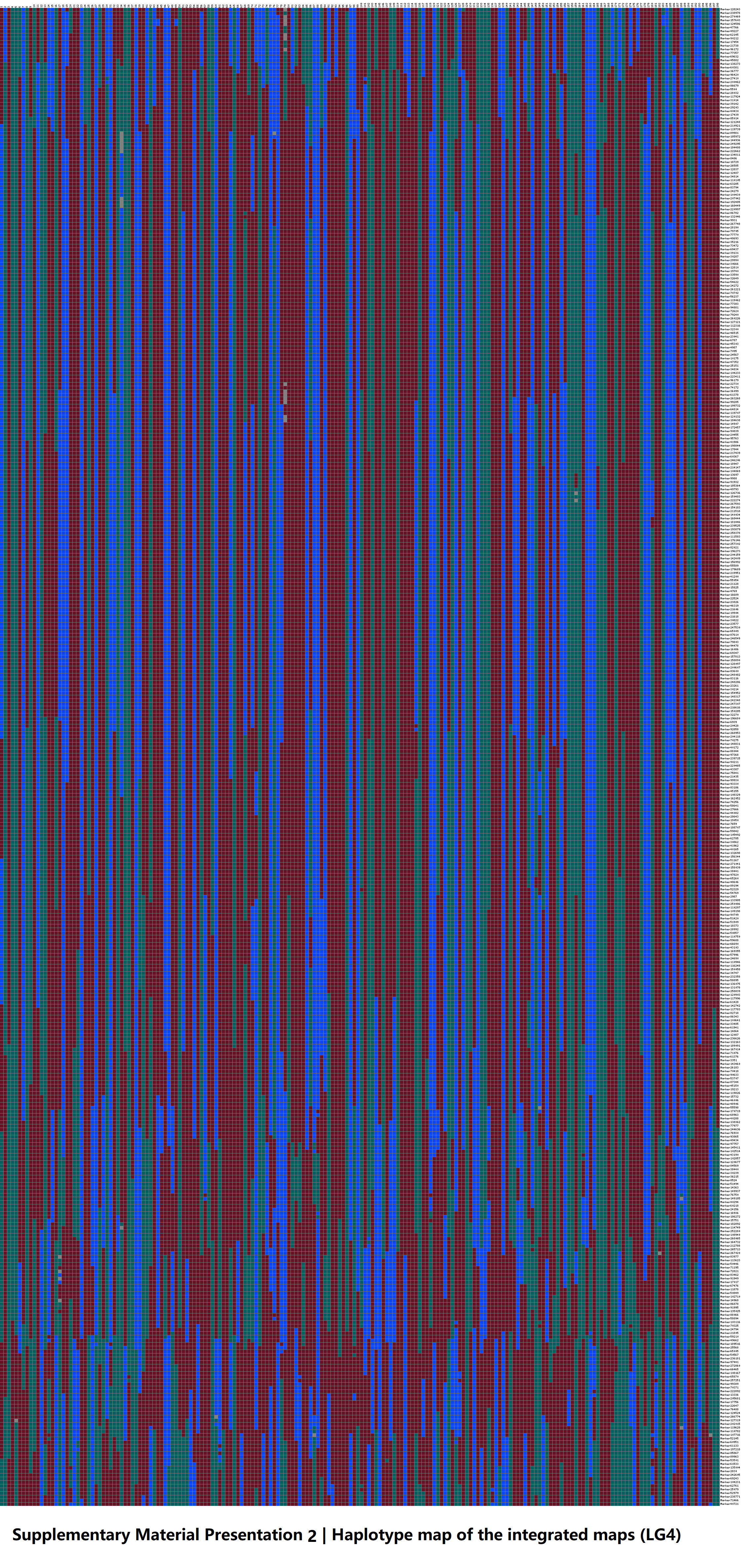

Supplement: Supplementary file 1 — Supplementary Information. [file 41598_2024_58167_MOESM1_ESM.zip › Supplementary material/Supplementary Material Presentation 2/LG4.haploMap.png]

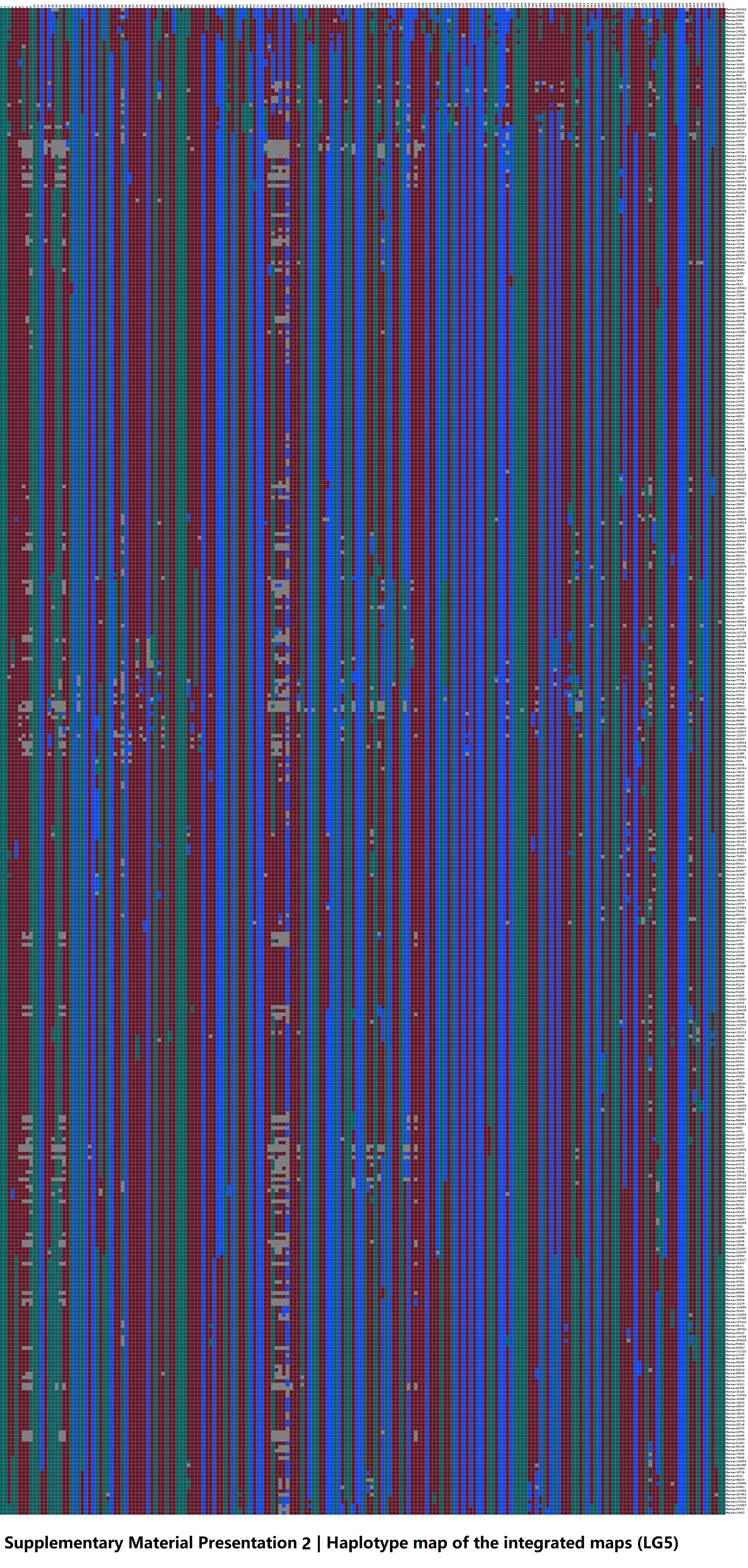

Supplement: Supplementary file 1 — Supplementary Information. [file 41598_2024_58167_MOESM1_ESM.zip › Supplementary material/Supplementary Material Presentation 2/LG5.haploMap.png]

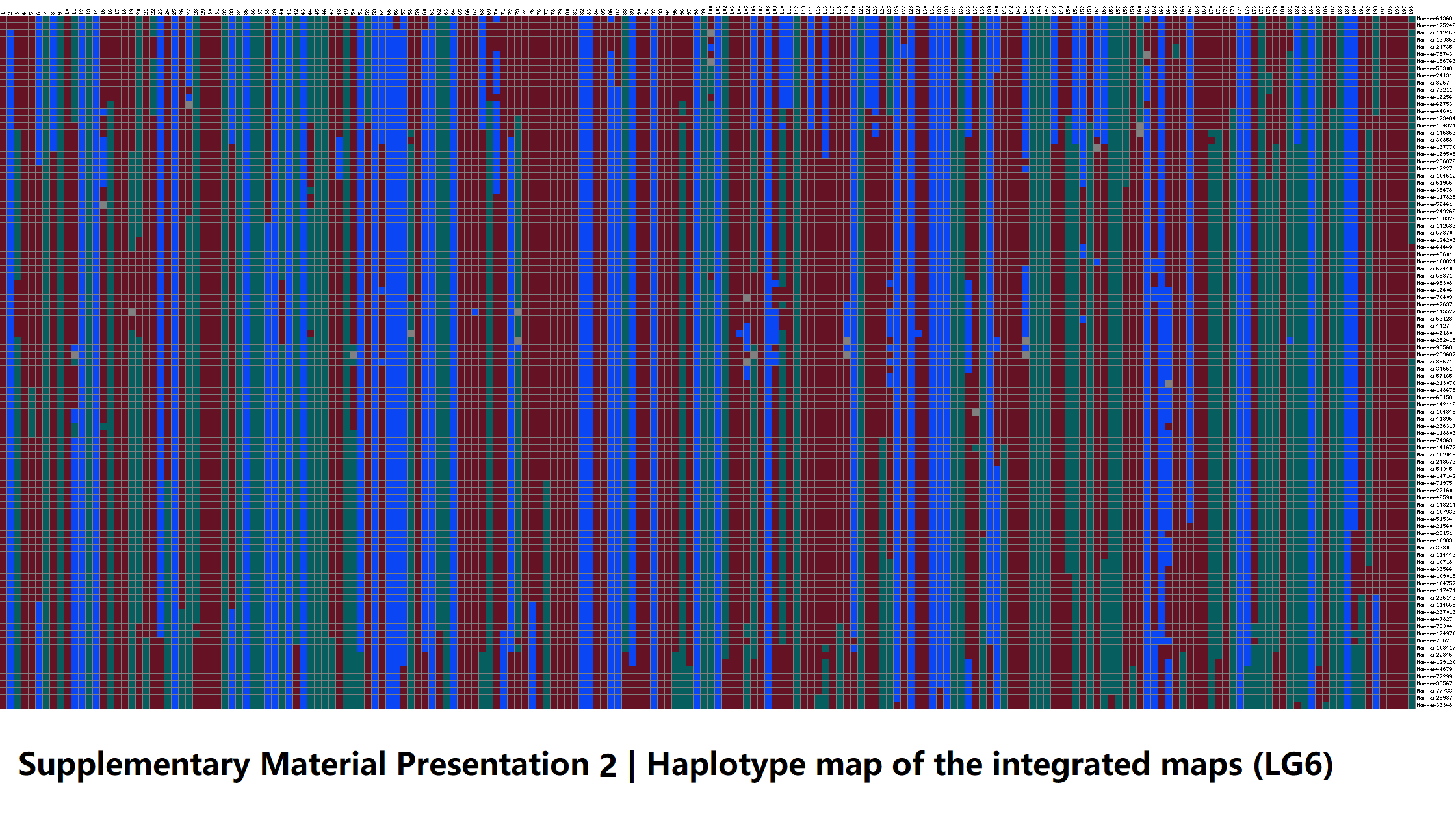

Supplement: Supplementary file 1 — Supplementary Information. [file 41598_2024_58167_MOESM1_ESM.zip › Supplementary material/Supplementary Material Presentation 2/LG6.haploMap.png]

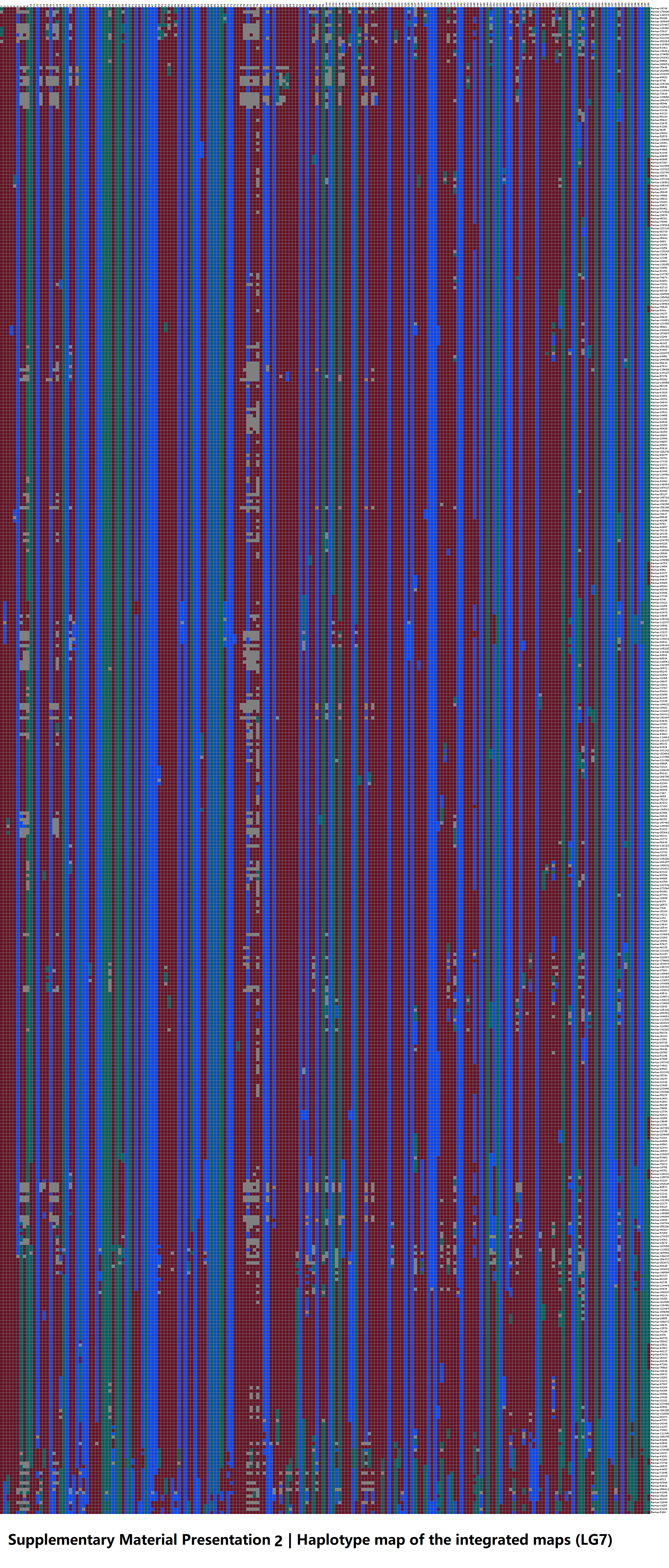

Supplement: Supplementary file 1 — Supplementary Information. [file 41598_2024_58167_MOESM1_ESM.zip › Supplementary material/Supplementary Material Presentation 2/LG7.haploMap.png]

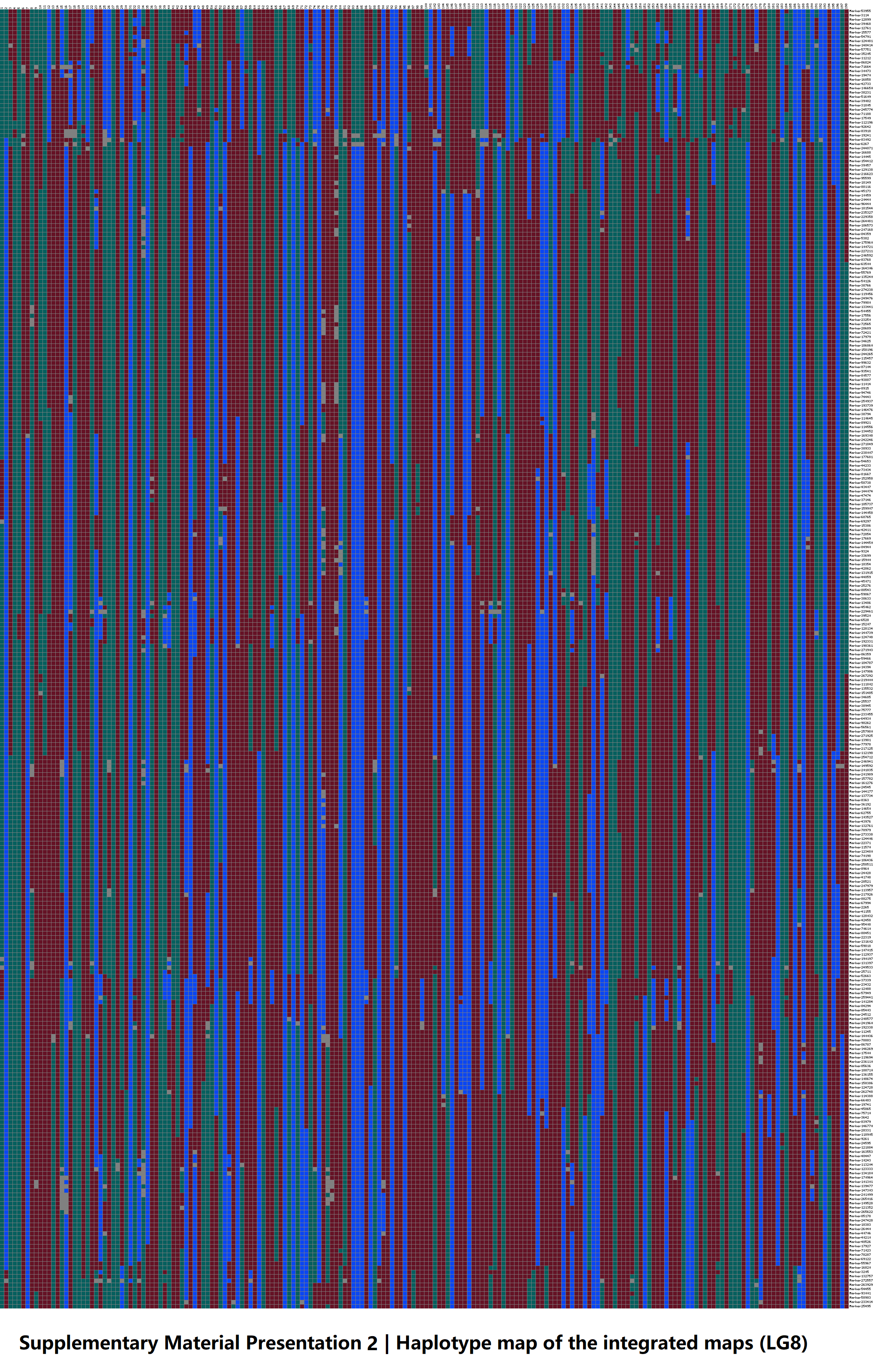

Supplement: Supplementary file 1 — Supplementary Information. [file 41598_2024_58167_MOESM1_ESM.zip › Supplementary material/Supplementary Material Presentation 2/LG8.haploMap.png]

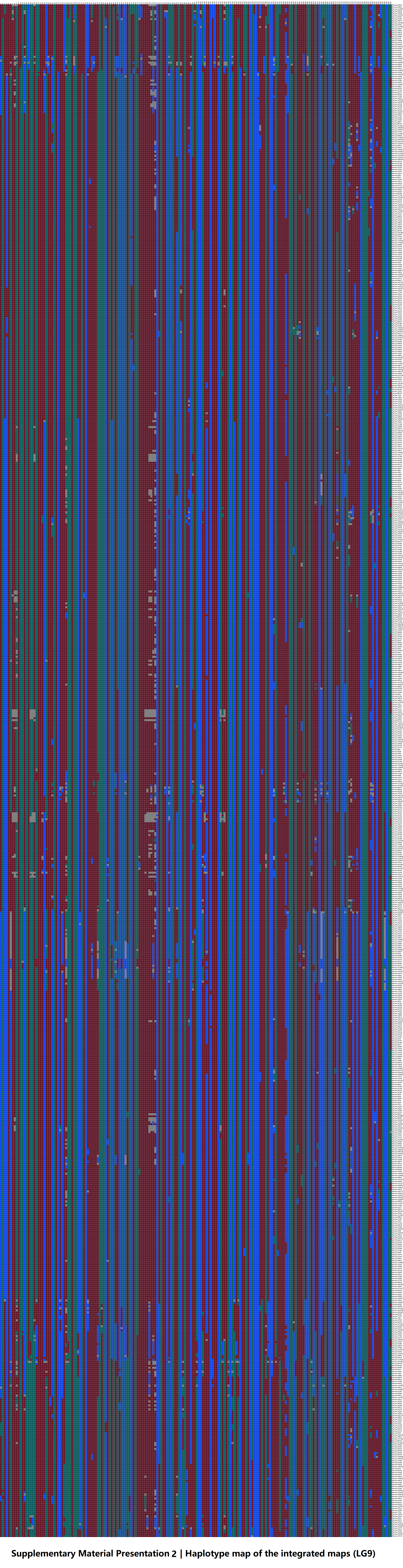

Supplement: Supplementary file 1 — Supplementary Information. [file 41598_2024_58167_MOESM1_ESM.zip › Supplementary material/Supplementary Material Presentation 2/LG9.haploMap.png]

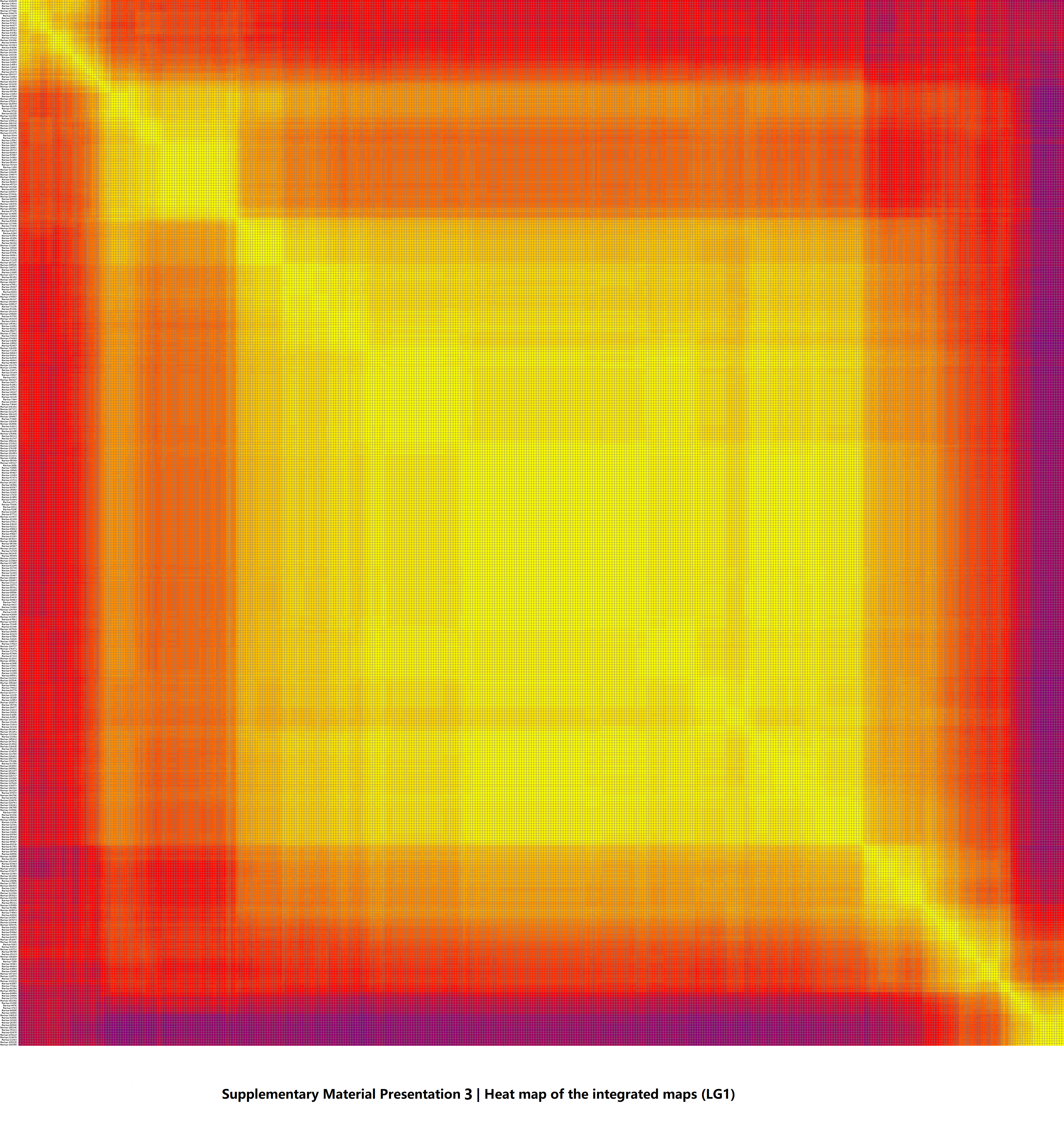

Supplement: Supplementary file 1 — Supplementary Information. [file 41598_2024_58167_MOESM1_ESM.zip › Supplementary material/Supplementary Material Presentation 3/LG1.heatMap.png]

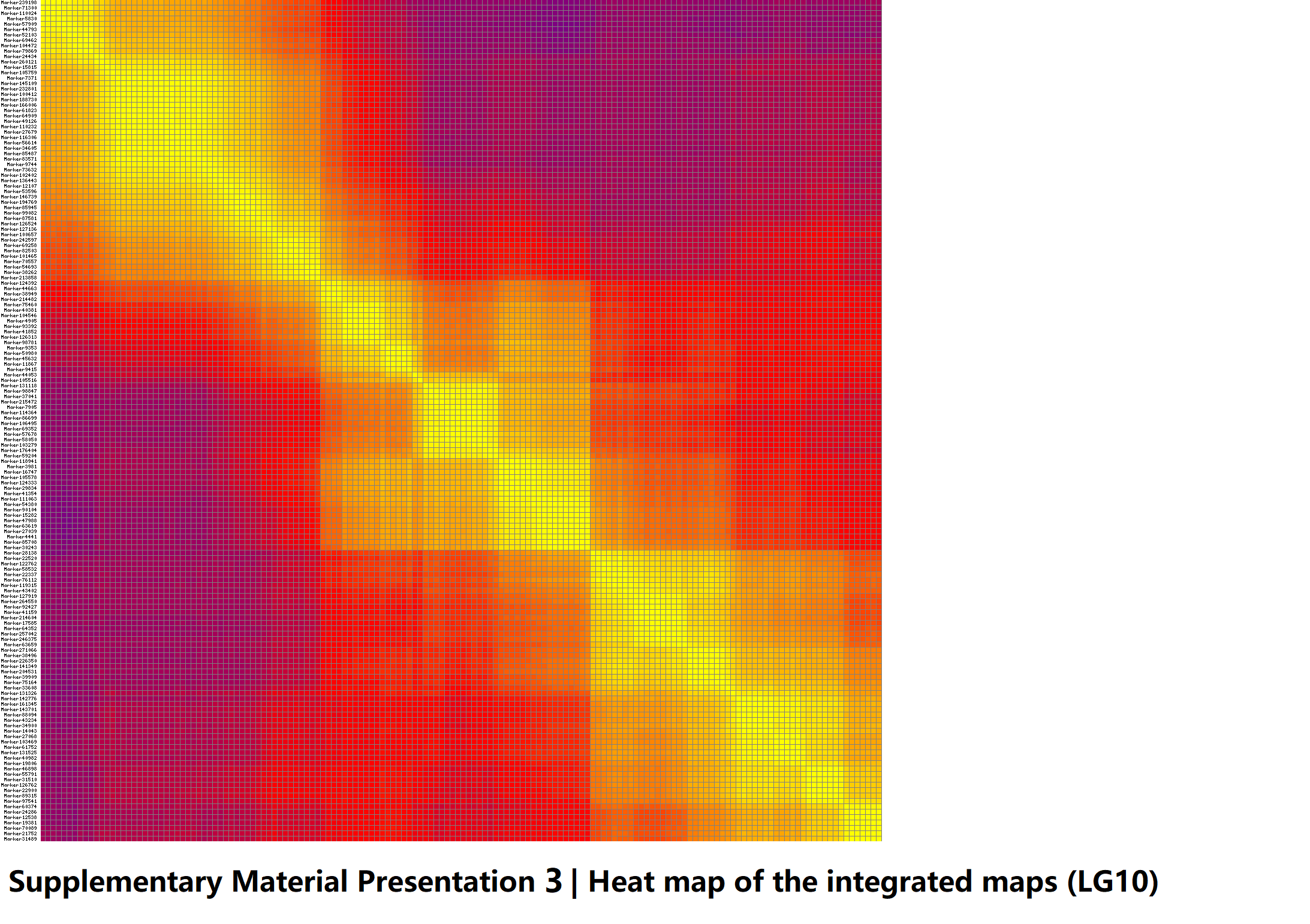

Supplement: Supplementary file 1 — Supplementary Information. [file 41598_2024_58167_MOESM1_ESM.zip › Supplementary material/Supplementary Material Presentation 3/LG10.heatMap.png]

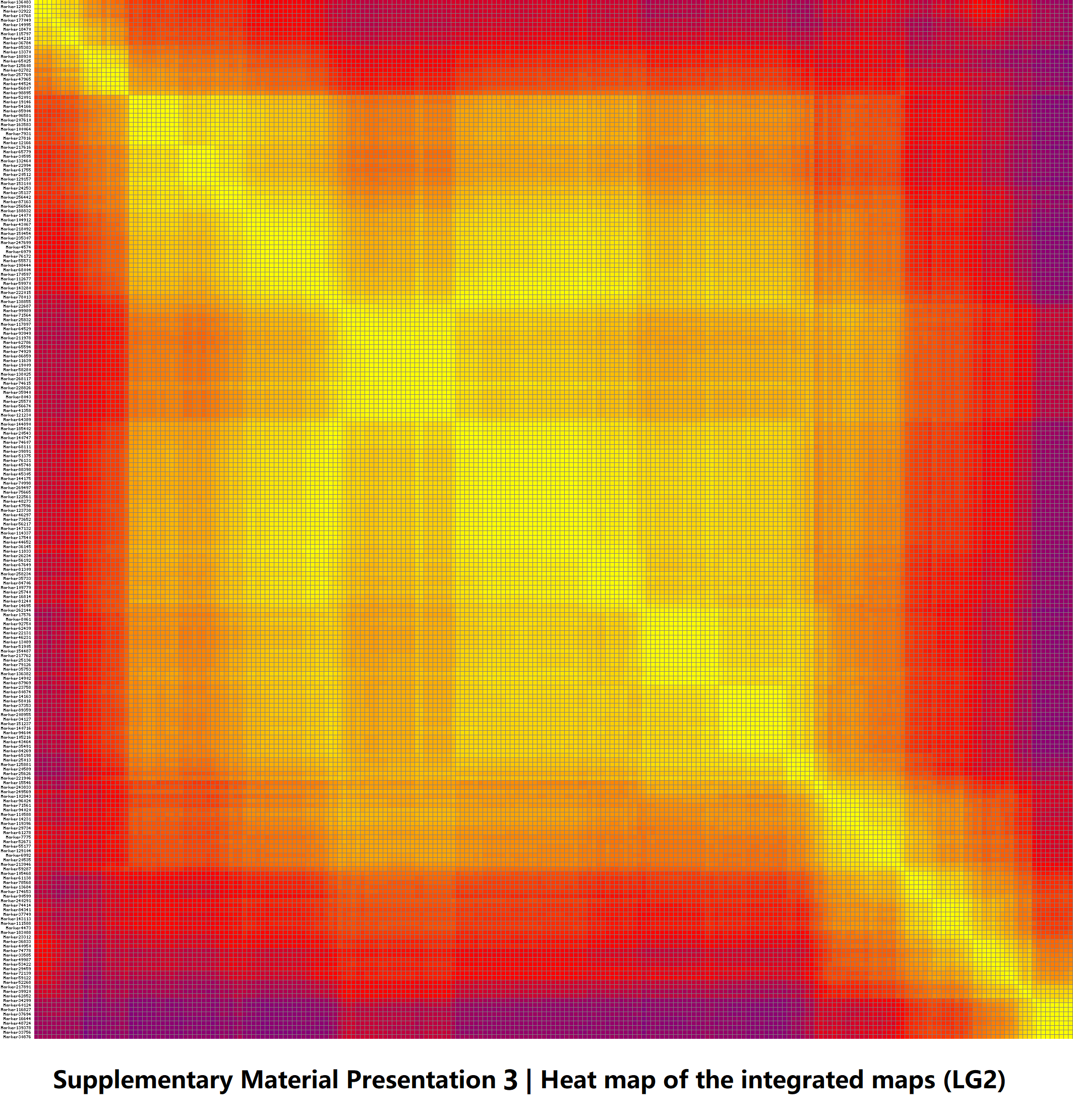

Supplement: Supplementary file 1 — Supplementary Information. [file 41598_2024_58167_MOESM1_ESM.zip › Supplementary material/Supplementary Material Presentation 3/LG2.heatMap.png]

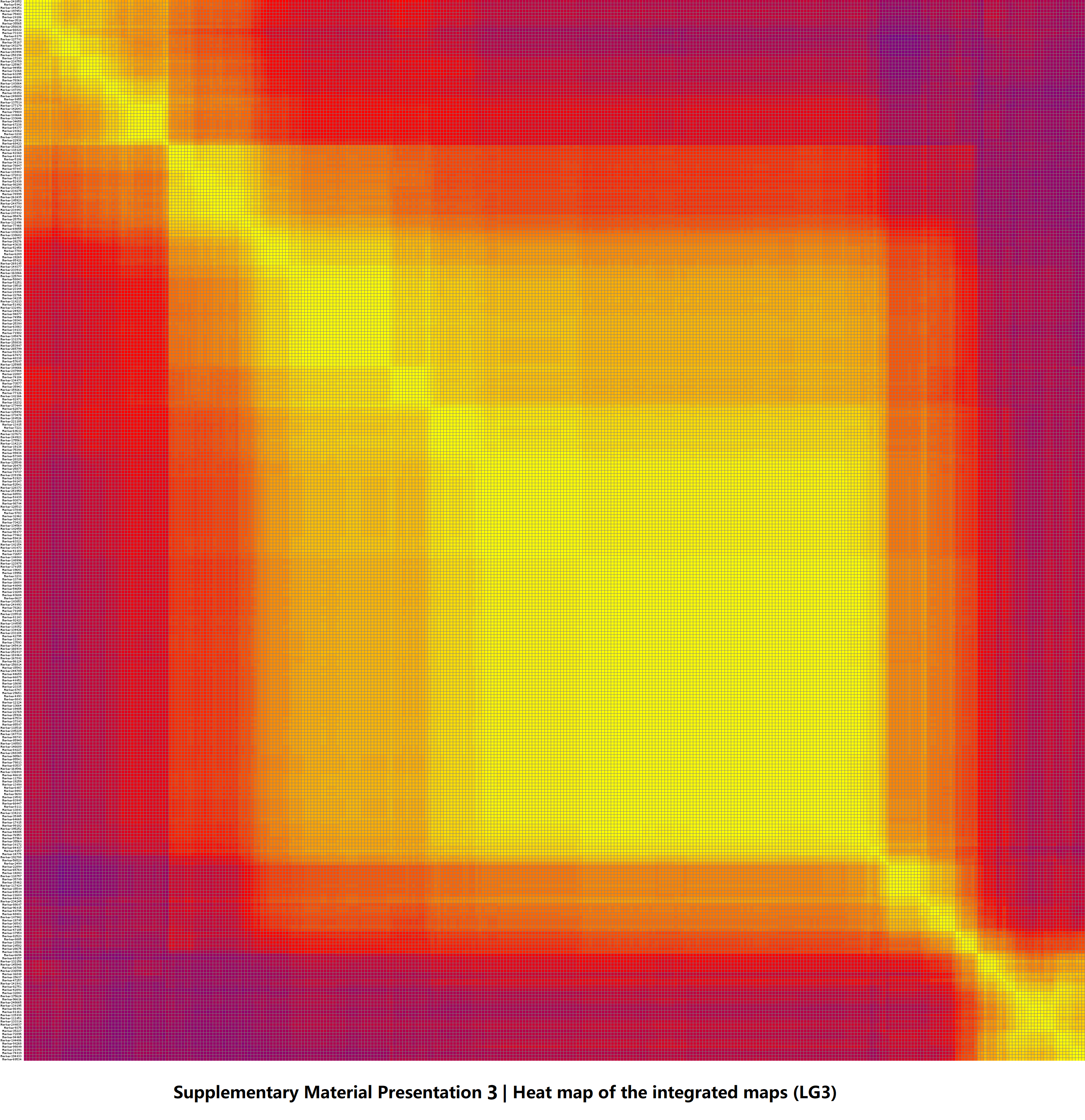

Supplement: Supplementary file 1 — Supplementary Information. [file 41598_2024_58167_MOESM1_ESM.zip › Supplementary material/Supplementary Material Presentation 3/LG3.heatMap.png]

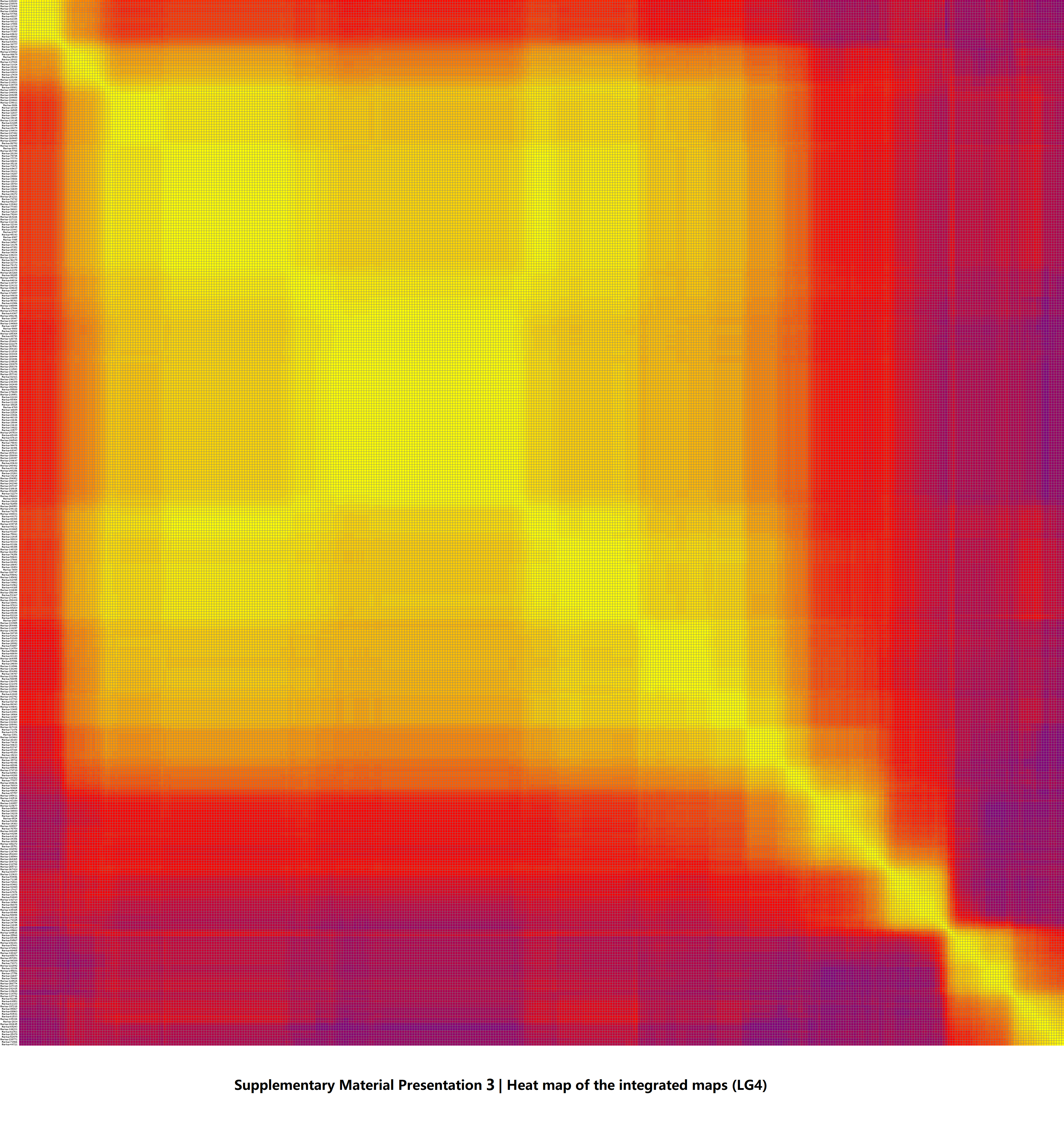

Supplement: Supplementary file 1 — Supplementary Information. [file 41598_2024_58167_MOESM1_ESM.zip › Supplementary material/Supplementary Material Presentation 3/LG4.heatMap.png]

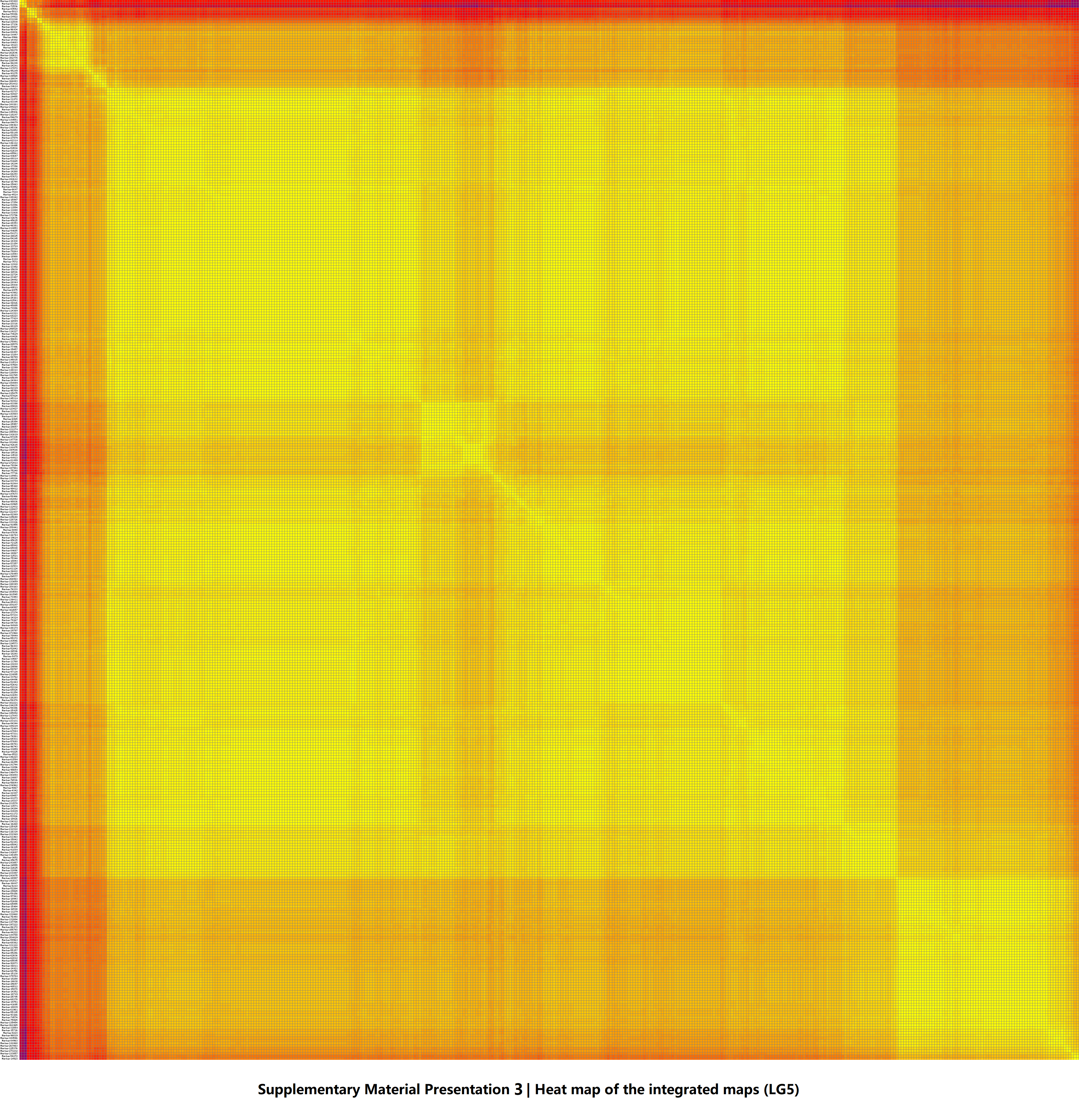

Supplement: Supplementary file 1 — Supplementary Information. [file 41598_2024_58167_MOESM1_ESM.zip › Supplementary material/Supplementary Material Presentation 3/LG5.heatMap.png]

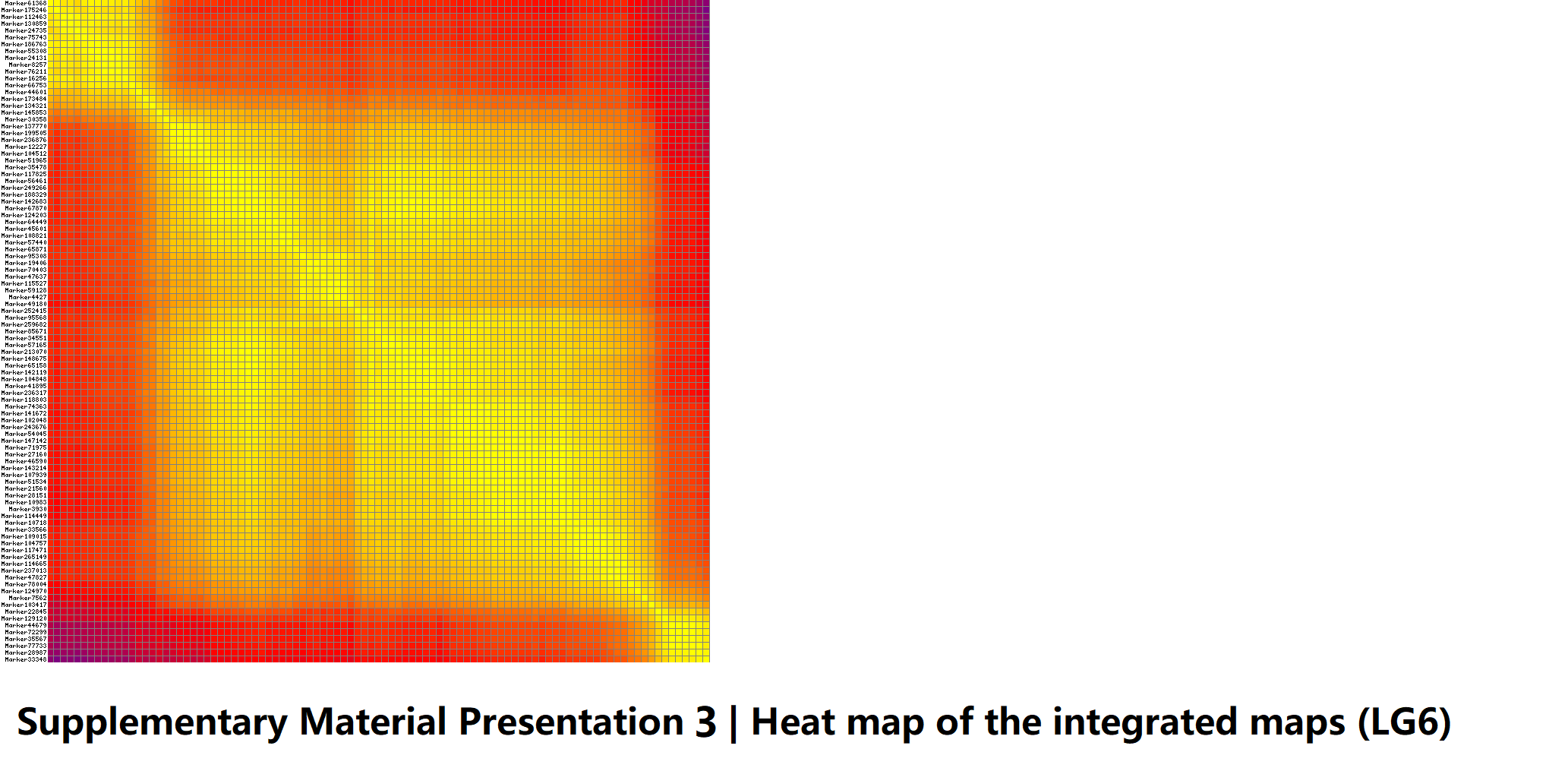

Supplement: Supplementary file 1 — Supplementary Information. [file 41598_2024_58167_MOESM1_ESM.zip › Supplementary material/Supplementary Material Presentation 3/LG6.heatMap.png]

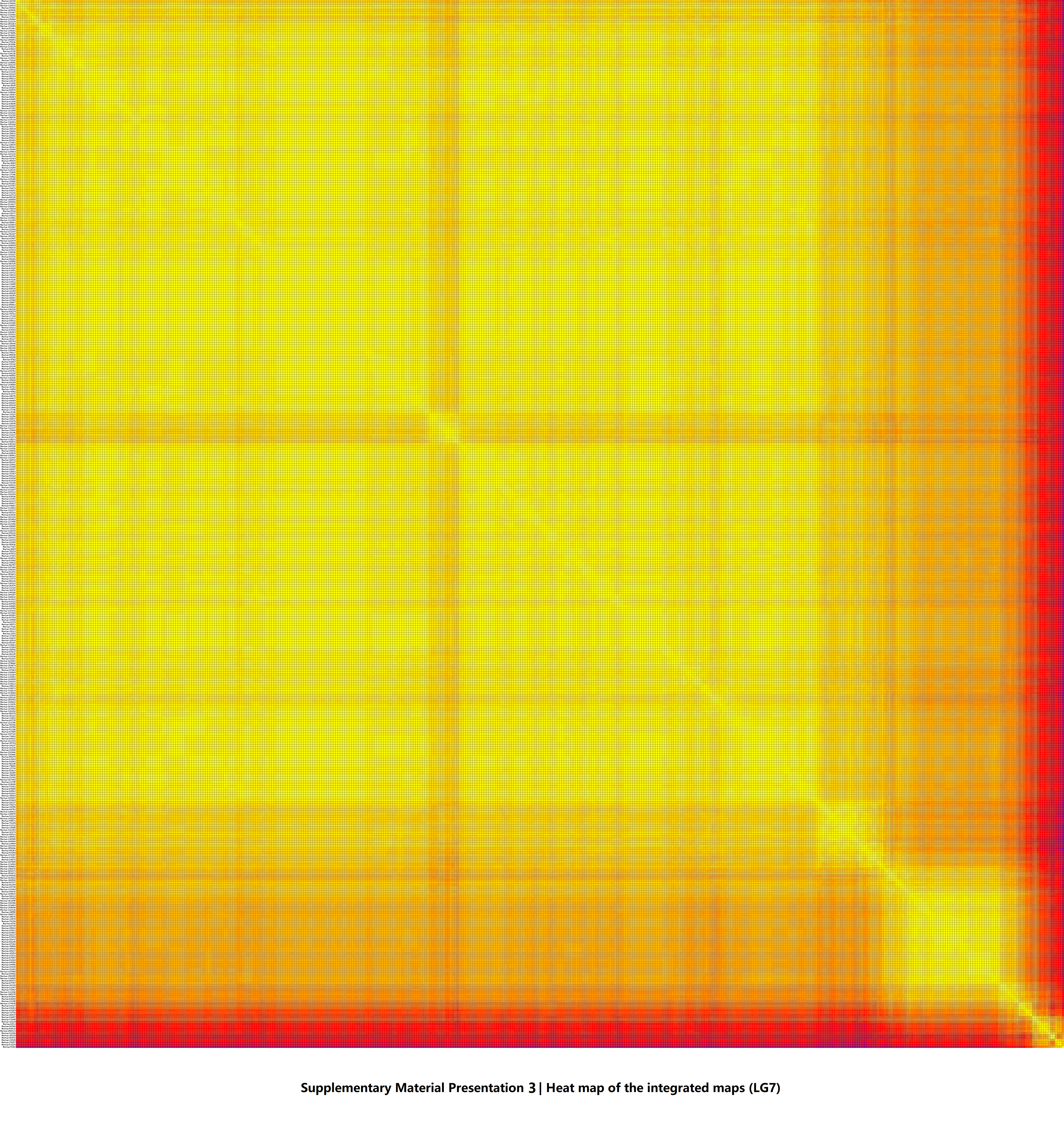

Supplement: Supplementary file 1 — Supplementary Information. [file 41598_2024_58167_MOESM1_ESM.zip › Supplementary material/Supplementary Material Presentation 3/LG7.heatMap.png]

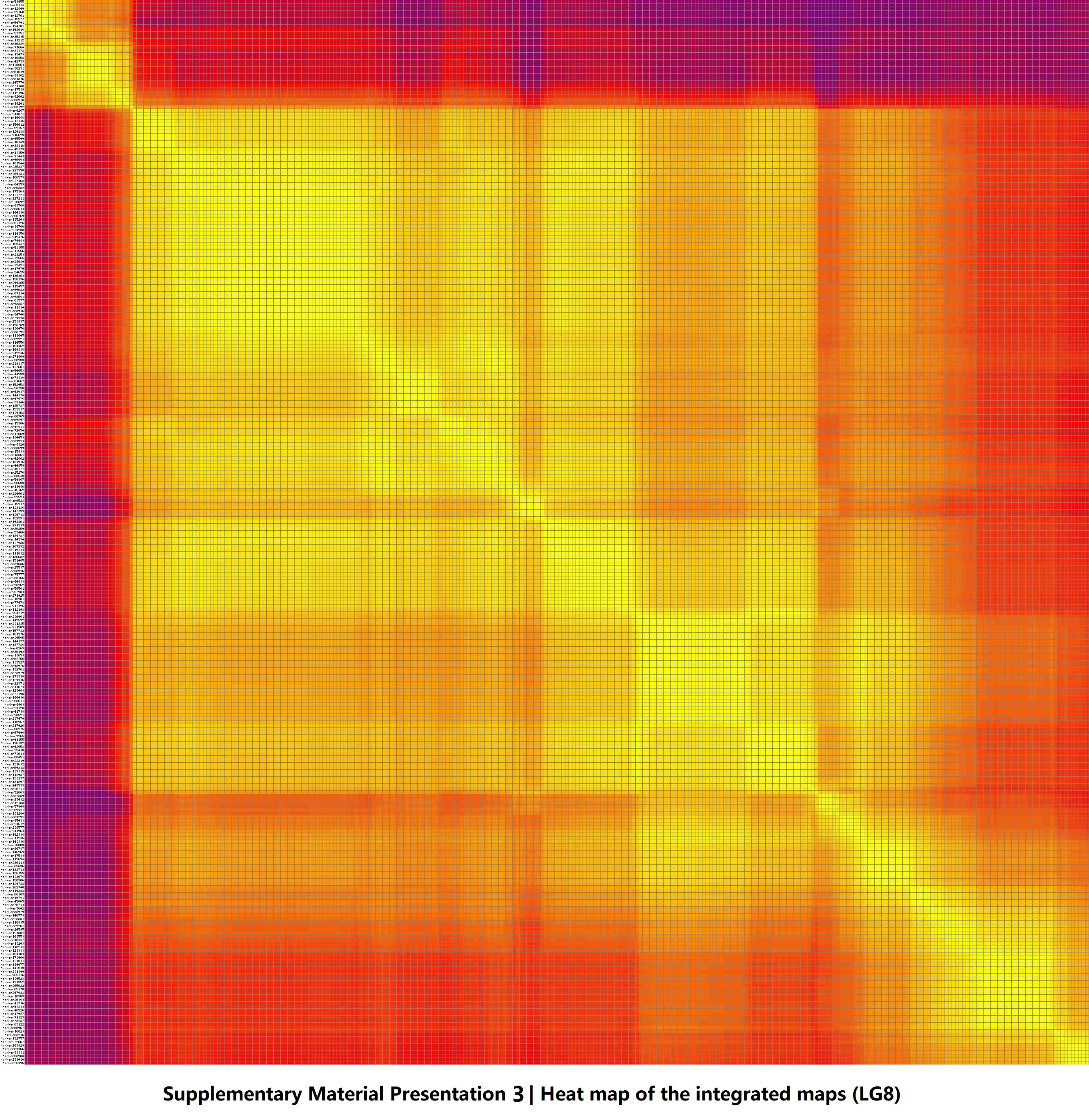

Supplement: Supplementary file 1 — Supplementary Information. [file 41598_2024_58167_MOESM1_ESM.zip › Supplementary material/Supplementary Material Presentation 3/LG8.heatMap.png]

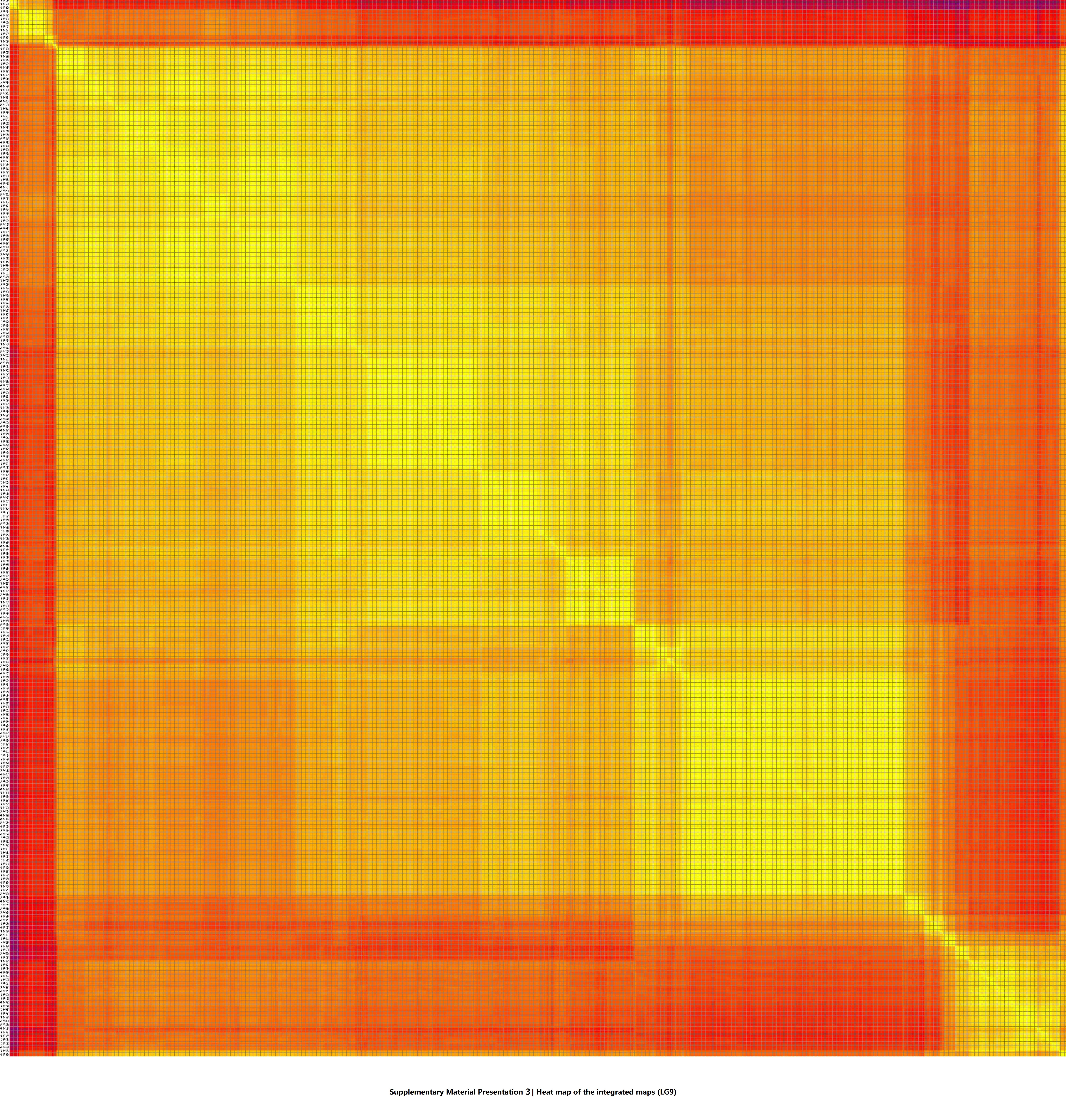

Supplement: Supplementary file 1 — Supplementary Information. [file 41598_2024_58167_MOESM1_ESM.zip › Supplementary material/Supplementary Material Presentation 3/LG9.heatMap.png]
