# Supplementary material for: Construction of a high-density genetic map based on large-scale marker development in Coix lacryma-jobi L. using specific-locus amplified fragment sequencing (slaf-seq)
Source: Sci Rep. 2024 Apr 26;14:9606. doi: 10.1038/s41598-024-58167-8 (PMC11053130; doi:10.1038/s41598-024-58167-8)
Supplement: Supplementary file 1 — Supplementary Information. [file 41598_2024_58167_MOESM1_ESM.zip › Supplementary material/Supplementary Material.doc]

**Supplementary Material**

**Supplementary Material Presentation 1 | Linkage group 1 to 10 of high-density linkage map for Coix lacryma-jobi L.** Segregation distortion markers on the map are highlighted in green.

**Supplementary Material Presentation 2 | Haplotype map of the integrated maps（LG1-LG10）.** Each row represents a marker. Markers are ranked in accordance with the map order. Each of the two columns represents an individual; blank columns are used between two individuals. The first and second columns represent the paternal and maternal chromosomes, respectively. The green and blue areas in the columns represent the first and second alleles from the parents, respectively. The white column represents the source of alleles that cannot be judged. The gray areas represent the deleted alleles .

**Supplementary Material Presentation 3 | Heat map of the integrated maps (LG1-LG10).** Markers of each row and column are ranked according to the map order; each small square represents the rate of recombination (r) between the two markers.

**Table S1 | Summary of the sequencing data.**

**Table S2 | SLAF markers and SNP loci on the map.** The SNPs within the SLAF marker allele sequences were marked in lowercase letters. The SLAF markers separated by linkage groups are shown in different sheets and the parent from which the alleles were derived are shown.
